# Lipoylation inhibition enhances radiation control of lung cancer by suppressing homologous recombination DNA damage repair

**DOI:** 10.1126/sciadv.adt1241

**Published:** 2025-03-12

**Authors:** Jui-Chung Chiang, Zengfu Shang, Tracy Rosales, Ling Cai, Wei-Min Chen, Feng Cai, Hieu Vu, John D. Minna, Min Ni, Anthony J. Davis, Robert D. Timmerman, Ralph J. DeBerardinis, Yuanyuan Zhang

**Affiliations:** ^1^ Department of Radiation Oncology, Harold C. Simmons Comprehensive Cancer Center, University of Texas Southwestern Medical Center, Dallas, TX 75390, USA.; ^2^ Howard Hughes Medical Institute, Eugene McDermott Center for Human Growth and Development, and Children’s Medical Center Research Institute, University of Texas Southwestern Medical Center, Dallas, TX 75235, USA.; ^3^ Peter O’Donnell, Jr. School of Public Health, University of Texas Southwestern Medical Center, Dallas, TX 75390, USA.; ^4^ Hamon Center for Therapeutic Oncology Research, Departments of Internal Medicine and Pharmacology, Harold C. Simmons Comprehensive Cancer Center, University of Texas Southwestern Medical Center, Dallas, TX 75390, USA.

## Abstract

Lung cancer exhibits altered metabolism, influencing its response to radiation. To investigate the metabolic regulation of radiation response, we conducted a comprehensive, metabolic-wide CRISPR-Cas9 loss-of-function screen using radiation as selection pressure in human non–small cell lung cancer. Lipoylation emerged as a key metabolic target for radiosensitization, with lipoyltransferase 1 (LIPT1) identified as a top hit. LIPT1 covalently conjugates mitochondrial 2-ketoacid dehydrogenases with lipoic acid, facilitating enzymatic functions involved in the tricarboxylic acid cycle. Inhibiting lipoylation, either through genetic LIPT1 knockout or a lipoylation inhibitor (CPI-613), enhanced tumor control by radiation. Mechanistically, lipoylation inhibition increased 2-hydroxyglutarate, leading to H3K9 trimethylation, disrupting TIP60 recruitment and ataxia telangiectasia mutated (ATM)–mediated DNA damage repair signaling, impairing homologous recombination repair. In summary, our findings reveal a critical role of LIPT1 in regulating DNA damage and chromosome stability and may suggest a means to enhance therapeutic outcomes with DNA-damaging agents.

## Teaser

Genetic screen finds lipoylation as a target to improve radiation success in lung cancer by blocking DNA damage repair.

## INTRODUCTION

One hundred million people live with cancer, and approximately half receive radiotherapy as a part of their cancer-directed therapies (*1*). Despite technological advances enabling precise radiation delivery, adequate tumor control is hindered by toxicities to normal tissue. Lung cancer therapy, in particular, is complicated by radiation’s narrow therapeutic index as it is a key curative treatment across multiple stages of the disease. The proximity of lung tumors to vital radiosensitive organs, including the lungs, heart, bronchus, esophagus, brachial plexuses, and spinal cord, restricts the delivery of curative doses. Consequently, lung cancer remains the deadliest cancer in the United States and worldwide. Efforts to directly target DNA damage repair pathways to enhance tumor radiosensitivity have yielded limited results due to the shared pathway with normal tissues. Currently, there are no Food and Drug Administration (FDA)–approved radiosensitizers, and the only FDA-approved medication designed to protect healthy tissues, amifostine, is severely limited by its requirement for intravenous administration and extensive side effects (*2*).

Radiation substantially modifies microenvironmental metabolism by altering nutrient and oxygen delivery through vascular damage, recruiting and activating immune populations, and rewiring metabolism to overcome radiation-induced reactive oxygen species and repair DNA damage (*3*). Recent research indicates that lung cancer exhibits altered metabolism associated with radiation resistance (*4*, *5*). Therefore, targeting metabolism is a promising area of investigation to improve radiation outcome. Longstanding interest in the Warburg effect and redox balance has linked radioresistance with increased production of lactate (via glycolysis) (*6*, *7*), increased glutathione (via glutamine metabolism) (*8*, *9*) and nicotinamide adenine dinucleotide phosphate hydrogen (NADPH) (via the pentose phosphate pathway) (*10*, *11*). However, these pathways are not readily targetable, and their relative importance compared to other, less studied metabolic pathways remains unclear.

To address this knowledge gap, we conducted an unbiased, metabolic-wide, CRISPR screen and unexpectedly found that lipoylation is a key metabolic vulnerability in lung cancer cells subjected to radiation. Lipoylation provides an essential cofactor, lipoic acid (LA), for 2-ketoacid dehydrogenases, which controls nutrient entry to the tricarboxylic acid (TCA) cycle at multiple points (*12*). Our top target, lipoyltransferase 1 (LIPT1), is responsible for the covalent transfer of LA onto these enzymes. The metabolic perturbations caused by LIPT1 deficiency have been well elucidated in cells, mice, and patients in the context of inborn errors of metabolism (*13*). The recent discovery of lipoylation as a mediator of cell death through cuproptosis has led to a renewed interest in this pathway and its role in various disease processes (*14*, *15*). Nevertheless, the role of lipoylation in cancer initiation and therapeutic responses is just beginning to be explored (*16*, *17*).

Lipoylation is targetable by a LA analog, CPI-613, also known as devimistat. This drug inhibits pyruvate dehydrogenase (PDH) and α-ketoglutarate dehydrogenase (KGDH) complexes by disrupting the lipoylation of these enzymes. CPI-613 has been tested clinically as an experimental anticancer drug and granted orphan drug designation by the US FDA. CPI-613 is well tolerated in patients with cancer, although it did not have efficacy as a single agent in small cell lung cancer (*18*) or improve survival outcomes compared to surgical approaches when combined with FOLFIRINOX in borderline resectable pancreatic cancer (*19*–*22*).

In this study, we sought to explore the mechanism and therapeutic potential of lipoylation inhibition as a radiosensitization strategy in lung cancer. Using cancer cell lines deficient in LIPT1 or treated with a lipoylation inhibitor, we confirmed a radiosensitization effect by lipoylation inhibition. Mechanistically, we found that inhibition of lipoylation leads to impaired, homology-dependent DNA damage repair and decreased chromosome stability.

## RESULTS

### Unbiased screen reveals lipoylation as a key metabolic vulnerability in radiation response of lung cancer

To identify metabolic vulnerabilities to ionizing radiation in NSCLC, we conducted a metabolic wide, CRISPR-Cas9 loss-of-function screen using ionizing radiation as a selection pressure. Before the screen, we compared multiple human NSCLC cell lines isolated from treatment-naïve patients with lung cancer, ultimately selecting H157 for its sensitivity to radiation therapy and puromycin, ease of lentiviral transduction, and low chromosome copy numbers, which facilitate complete genetic knockouts.

**Fig. 1.**
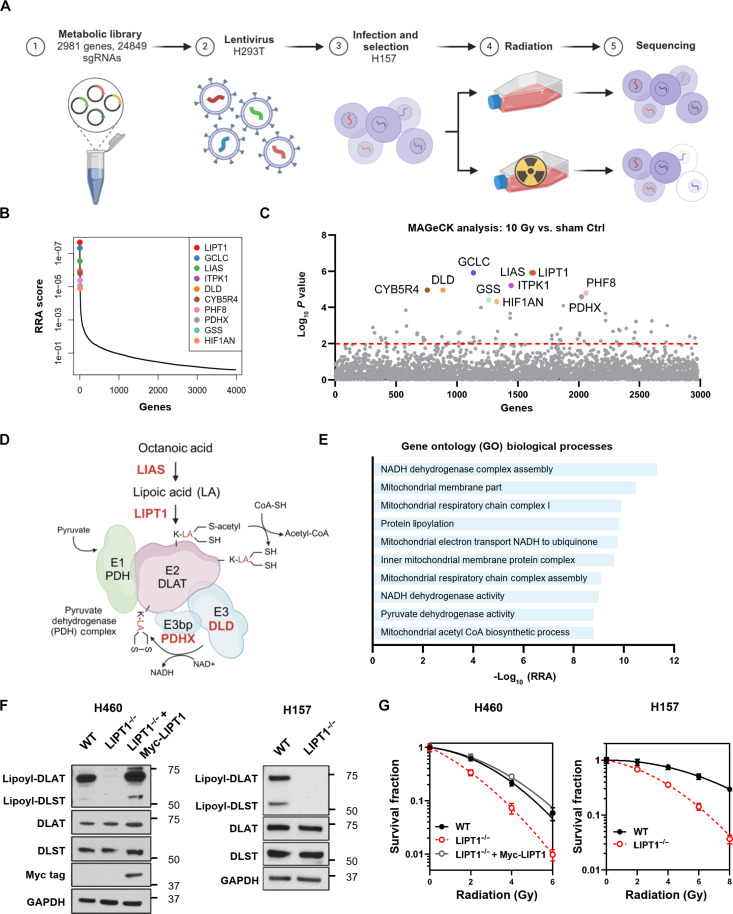
Metabolic CRISPR screen identifies lipoylation as metabolic vulnerability for radiation response in NSCLC. (**A**) Schematic illustration of CRISPR-Cas9 screen workflow. (**B**) Top 10 negatively selected genes ranked by robust rank aggregation (RRA) score in 10 Gy–irradiated group compared to the control. (**C**) Manhattan plot of the entire 2981 metabolic genes by log_10_ *P* value. The top 10 negatively selected genes are highlighted. (**D**) Schematic illustration of the function of LIPT1, LIAS, DLD, and PDHX, the four top hits involved in lipoylation. (**E**) Pathway analysis of the 10 most significantly depleted metabolic pathways by Gene Ontology classification in 10 Gy–irradiated cells compared to the nonirradiated cells. (**F**) Immunoblots of total and lipoylated PDH and α-KGDH subunits (DLAT and DLST, respectively). Glyceraldehyde-3-phosphate dehydrogenase (GAPDH), DLAT, and DLST were blotted as loading controls. (**G**) Clonogenic assay of indicated cell lines after 2, 4, 6, and 8 Gy IR. The surviving fraction was normalized to the corresponding sham control, and survival curves were fitted using the linear-quadratic model.

The overall schema of the screen is shown in Fig. 1A and results in data S1. Briefly, H157 cells were transduced with lentivirus containing the single-guide RNA (sgRNA) library at a low multiplicity of infection (MOI), with 24,849 sgRNAs (including 1000 noncoding controls) targeting 2981 genes. After puromycin selection, cells with adequate coverage for each guide (at least 1000×) were subjected to either sham or 10 gray (Gy) ionizing radiation (IR), with five biological replicates each. The cells were collected after 14 days in culture to allow time for cell death. Genomic DNA was then extracted, and guide RNAs (gRNAs) were amplified by polymerase chain reaction (PCR) and sequenced. All samples retained approximately 99 to 100% representation of the library. Using Model-based Analysis of Genome-wide CRISPR-Cas9 knockout (MAGeCK), we identified genes whose guides were most depleted in the irradiated cells compared to the control, indicating that silencing these genes resulted in decreased survival after radiation.

Radiation kills tumors by causing DNA double-strand breaks (DSBs) either through direct ionization or indirectly via reactive oxygen species, with two-thirds of DSBs resulting from the latter. As expected, metabolic players involved in redox balance and DNA damage repair were among the most depleted (Fig. 1, B and C). Among the top 10 targets, *GCLC* and *GSS* encode enzymes responsible for glutathione synthesis, which forms the largest antioxidant pool in mammalian cells and is linked to radiation response (*9*). *CYB5R4* encodes a member of cytochrome b5 reductase family, which regulates redox balance and DNA damage response (*23*). IPTK1 (inositol-tetrakisphosphate 1-kinase) is involved in inositol hexaphosphate (IP6) synthesis, which can activate nonhomologous end joining (NHEJ) by binding to the DNA-PK complex and changing its conformation (*24*). PHF8 (PHD finger protein 8) plays a role in genomic stability and tumorigenesis (*25*), while HIF1AN (hypoxia inducible factor 1 subunit alpha inhibitor) regulates the transcriptional activity of hypoxia-inducible factor (*26*). Although their therapeutic potential as radiosensitization targets is not fully understood, these genes represent expected targets from a successful radiation screen based on their known cellular functions and known mechanism of radiation response.

Unexpectedly, four genes involved in protein lipoylation and 2-ketoacid dehydrogenase activity were among the top 10 targets in our screen (Fig. 1, B to D): LIPT1, LA synthase (LIAS), dihydrolipoamide dehydrogenase (DLD), and PDH complex component X (PDHX). Lipoylation, the covalent conjugation of LA to a protein, is essential for the enzymatic function of the PDH complex [PDH, containing DLAT (dihydrolipoamide S-acetyltransferase), and PDHX], the α-KGDH complex [α-KGDHC, containing DLST (dihydrolipoamide S-succinyltransferase)], the branched-chain alpha-keto acid dehydrogenase complex (BCKDHC, containing DBT), and the glycine cleavage system (containing GCSH). Deficient lipoylation can obstruct substrate entry into the TCA cycle at multiple points through inhibition of the 2-ketoacid dehydrogenases PDH, α-KGDHC, and BCKDHC (*12*, *27*). LIAS synthesizes LA from octanoic acid in the mitochondria, and LIPT1 covalently conjugates LA to the E2 subunit of 2-ketoacid dehydrogenases. DLD is the common E3 subunit of mitochondrial 2-ketoacid dehydrogenases, and PDHX (E3 binding protein) is a known target of lipoylation on PDH (Fig. 1D).

To identify metabolic vulnerabilities at the pathway level, we assigned each sgRNA to metabolic pathways based on Gene Ontology classification and performed MAGeCK analysis (Fig. 1E). We found that the top 10 depleted pathways involved lipoylation and related mitochondrial activities including PDH and the electron transport chain.

To validate the results from the CRISPR screen, we established stable LIPT1 knockout (LIPT1^−/−^) and nontargeting control wild-type (WT) NSCLC H460 and H157 cells using CRISPR-Cas9. Consistent with previous reports (*13*), LIPT1^−/−^ cells showed decreased lipoylated DLAT (E2 subunit of PDH) and DLST (E2 subunit of α-KGDH), which could be rescued by stably expressing exogenous Myc-tagged LIPT1. The overall expression of DLAT and DLST was similar between WT and LIPT1^−/−^ cells (Fig. 1F). We next performed clonogenic assays under various doses of radiation and found that LIPT1^−/−^ cells were more sensitive to radiation. This sensitivity was abolished by expressing exogenous LIPT1 (Fig. 1G and fig. S1). Together, these findings highlight lipoylation as a key metabolic vulnerability in the radiation response of lung cancer cells.

### Depletion of LIPT1 inhibits DNA damage repair and enhances chromosome instability

**Fig. 2.**
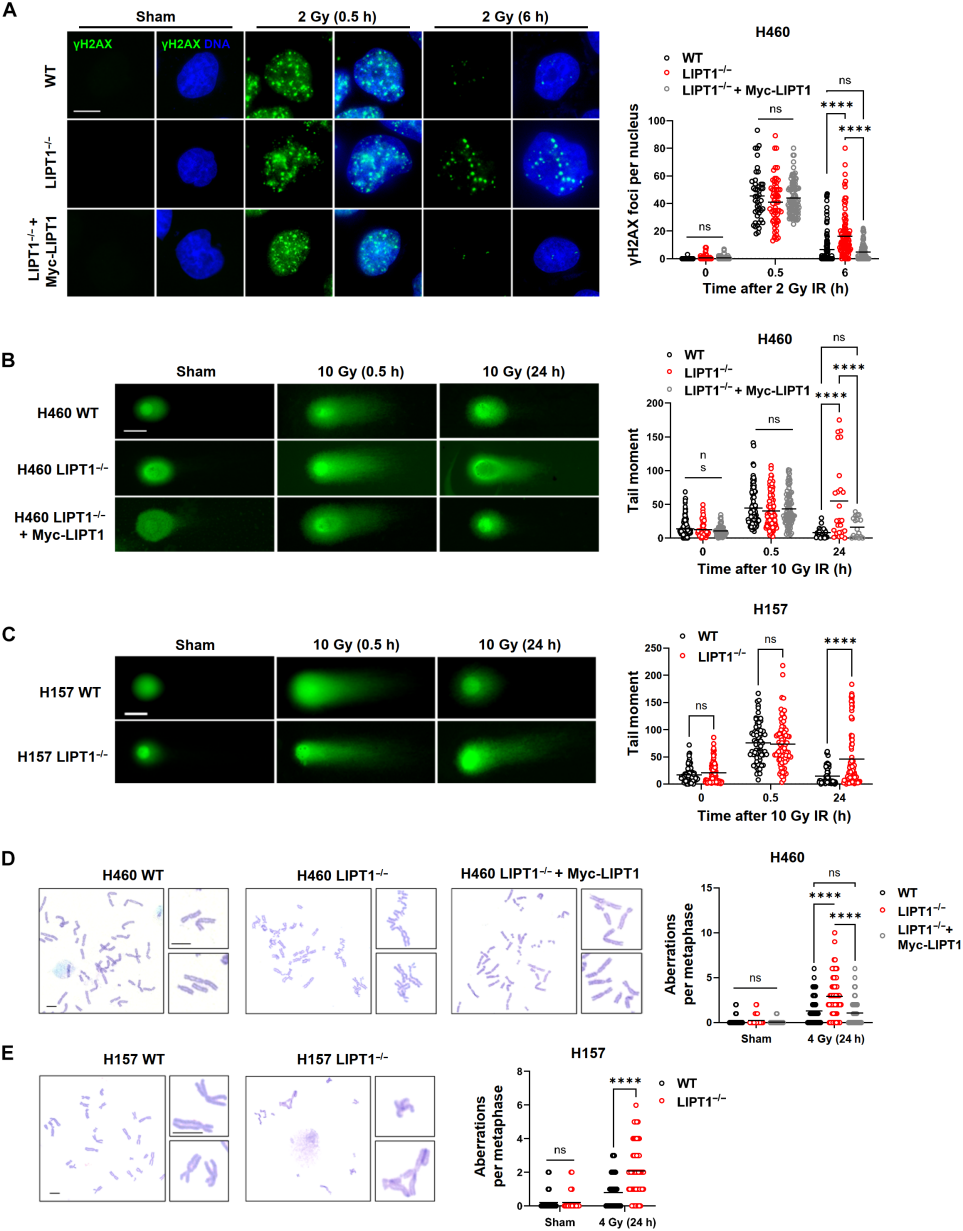
LIPT1 regulates DNA damage repair and chromosome stability. (**A**) Representative images and quantification of γH2AX foci by immunofluorescence staining in nonirradiated cells and at 0.5 and 6 hours (h) after 2-Gy IR in WT, LIPT1^−/−^ H460, and LIPT1^−/−^ H460 stably expressing Myc-LIPT1 cells. Nuclei were stained with Hoechst 33342. Scale bar, 10 μm. (**B**) Representative images and quantification of tail moment at specified time points after 10 Gy by neutral comet assay for the evaluation of DSBs in WT, LIPT1^−/−^ H460, and LIPT1^−/−^ H460 stably expressing Myc-LIPT1 cells. Scale bar, 50 μm. (**C**) Representative images and quantification of tail moment at specified time points after 10Gy by neutral comet assay for the evaluation of DSBs in WT and LIPT1^−/−^ H157 cells. Scale bar, 50 μm. (**D**) Representative images of metaphases and quantification of chromosome aberrations on mitotic chromosomes by chromosome spread assay at 24 hours post 4-Gy IR in WT, LIPT1^−/−^ H460, and LIPT1^−/−^ H460 stably expressing Myc-LIPT1 cells. Scale bar, 5 μm. (**E**) Representative images of metaphases and quantification of chromosome aberrations on mitotic chromosomes by chromosome spread assay at 24 hours post 4-Gy IR in WT and LIPT1^−/−^ H157 cells. Scale bar, 5 μm. For (A) to (E), imaging and quantification were performed on 60 to 100 cells per treatment. Two-way analysis of variance (ANOVA) was used for the statistical analyses. ****P < 0.0001. ns, not significant.

To assess the cellular response to ionizing radiation in WT and LIPT1^−/−^ cells, we examined the dynamics of the DNA damage marker γH2AX foci at various intervals after 2-Gy IR. Following DNA DSBs, rapid phosphorylation of S139 of H2AX molecules adjacent to the break site results in the formation of γH2AX foci, serving as a sensitive indicator of DSBs. Notably, while no differences in γH2AX foci were evident immediately after radiation among experimental groups, a sustained increase was observed at later time points in LIPT1^−/−^ cells compared to controls (Fig. 2A).

To confirm that the γH2AX foci represent unrepaired DNA DSBs, we conducted neutral comet assays to visualize DNA DSBs at the single-cell level both immediately and 24 hours after 10 Gy using single-cell electrophoresis. The tail moment, measuring both the percentage of DNA content in the tail and the tail length, revealed similar levels of DSBs at 30 min after radiation but elevated DSB levels at 24 hours post-IR in both LIPT1^−/−^ NSCLC cell lines (Fig. 2, B and C). These results indicate that LIPT1 deficiency impairs DNA DSB repair.

Cytogenetic analysis of metaphase spreads further corroborated these findings, revealing an increase in chromosome aberrations, including breaks, deletions, and chromatid interchanges (*28*), in LIPT1^−/−^ compared to the WT (Fig. 2, D and E) in both H460 and H157 cells. These observations underscore the critical role of LIPT1 in preserving chromosomal integrity and facilitating DNA damage repair, contributing to the radiosensitization effect observed under lipoylation inhibition.

### Metabolomic profiling of LIPT1^−/−^ H460 cells reveals decreased abundance of TCA intermediates and increased 2HG

**Fig. 3.**
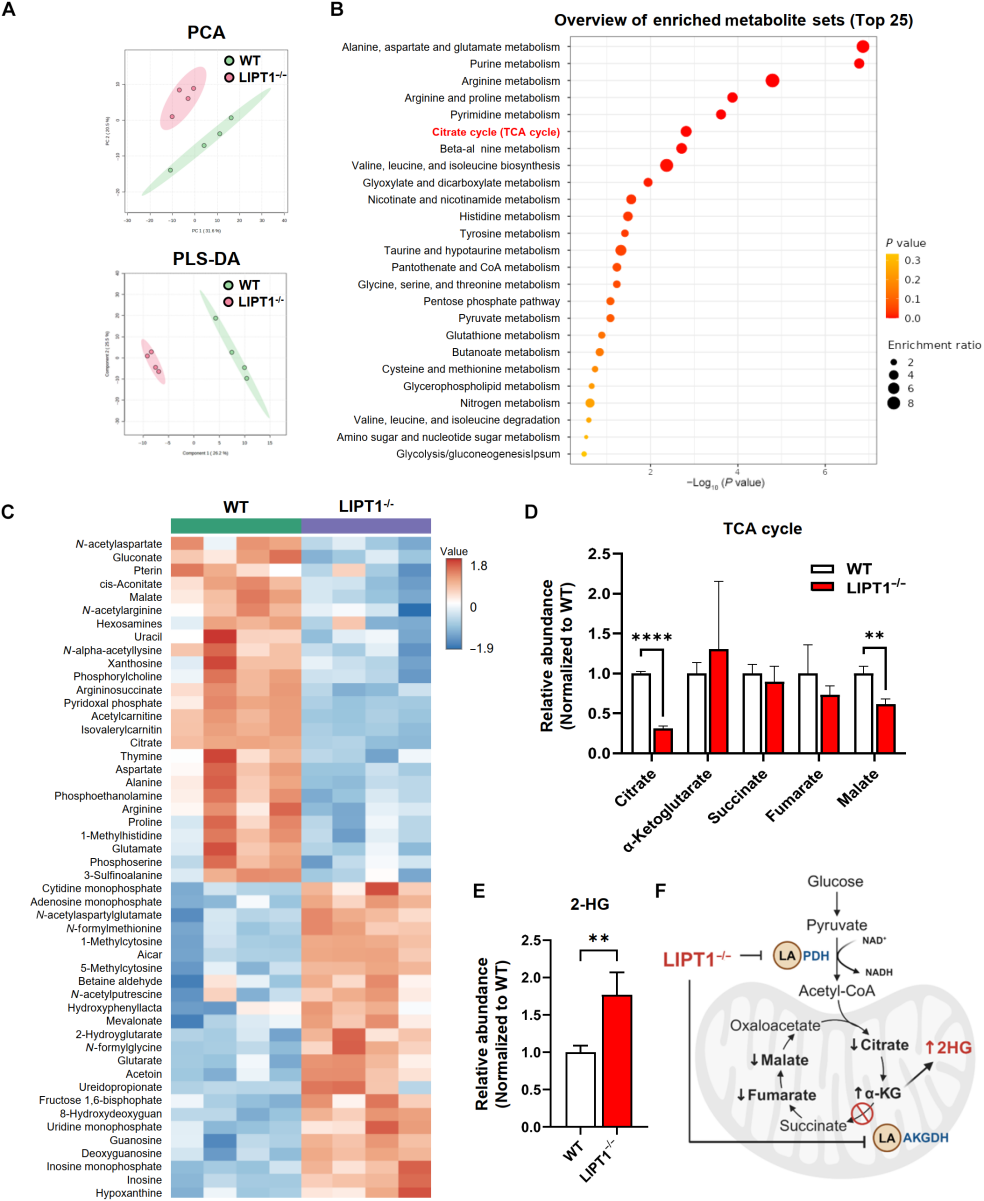
Metabolomic profiling of LIPT1^−/−^ H460 cells reveals decreased abundance of TCA intermediates and increased 2HG. (**A**) Principal components analysis (PCA) and partial least squares discriminant analysis (PLS-DA) of metabolomic profiles in WT and LIPT1^−/−^ H460 cells. (**B**) Metabolite set enrichment analysis comparing WT and LIPT1^−/−^ H460 cells. (**C**) Heatmap analysis of top 50 differential metabolites WT and LIPT1^−/−^ H460 cells. (**D**) Relative abundance of the indicated TCA cycle metabolites in WT and LIPT1^−/−^ H460 cells. (**E**) Relative abundance of 2-hydroxyglutarate (2HG) in WT and LIPT1^−/−^ H460 cells. (**F**) Impact of LIPT1 deficiency on enzymes and metabolites related to the TCA cycle. For (D) to (E), error bars represent SD. Unpaired *t* tests were used for the statistical analyses. ****P < 0.0001, **P < 0.01.

To investigate the metabolic perturbations potentially contributing to increased DNA damage and chromosome instability in LIPT1^−/−^ cells, we conducted unbiased metabolomic analyses on WT and LIPT1^−/−^ H460 cells (Fig. 3). Principal components analysis and partial least squares discriminant analysis revealed distinct metabolic perturbations in LIPT1^−/−^ H460 cells compared to WT controls (Fig. 3A).

Pathway enrichment analysis indicated that the most perturbed pathways included alanine, aspartate, and glutamate metabolism; nucleotide metabolism; arginine metabolism; and the TCA cycle (Fig. 3B). The most substantially altered metabolites are depicted in the heatmap (Fig. 3C). Multiple TCA cycle intermediates, including citrate and malate, decreased in abundance (Fig. 3D), while 2HG increased in LIPT1^−/−^ cells (Fig. 3E), as previously described (*13*). 2HG accumulation is thought to result from impaired α-KG oxidation, compounded by enhanced L-2HG formation by malate dehydrogenase and lactate dehydrogenase (Fig. 3F) (*29*, *30*). These results confirmed the expected metabolic perturbations from LIPT1 knockout.

Emerging evidence suggests that metabolites related to the TCA cycle play crucial roles in DNA damage repair (*31*). Succinate, fumarate, and 2HG are reported to serve as oncometabolites, competitively inhibiting α-KG–dependent dioxygenases, including histone lysine demethylases and other epigenetic modifiers to impair DNA damage repair (*32*, *33*). The TCA cycle also modulates DNA damage repair by maintaining histone acetylation via acetyl CoA (*34*); regulating NAD^+^ levels required for poly(adenosine diphosphate–ribose) polymerase activity (*35*); providing NADPH to protect against oxidative DNA damage (*36*); supporting de novo nucleotide synthesis (*37*); and supplying adenosine triphosphate (ATP) to fuel the repair process (*38*). Therefore, we postulate that lipoylation potentially affects DNA damage repair through multiple mechanisms, including chromatin remodeling, redox balance, and the availability of nucleotides and ATP.

### LIPT1^−/−^ cells exhibit elevated baseline H3K9me3 and impaired TIP60 recruitment to DNA damage sites

Chromatin remodeling allows the area around DNA lesions to become more accessible for DNA damage response and repair proteins (*39*). Recent studies have linked elevated 2HG with defective DSBs repair (*32*, *33*, *40*). 2HG competitively inhibits α-KG–dependent lysine demethylases, resulting in aberrant hypermethylation of histone 3 lysine 9 (H3K9) at loci surrounding DNA breaks. This hypermethylation masks a local H3K9 trimethylation signal crucial for the recruitment of TIP60, a histone acetyltransferase that acetylates ATM (ataxia telangiectasia–mutated) kinase, subsequently inducing ATM kinase autophosphorylation and its downstream signaling (*41*–*43*).

**Fig. 4.**
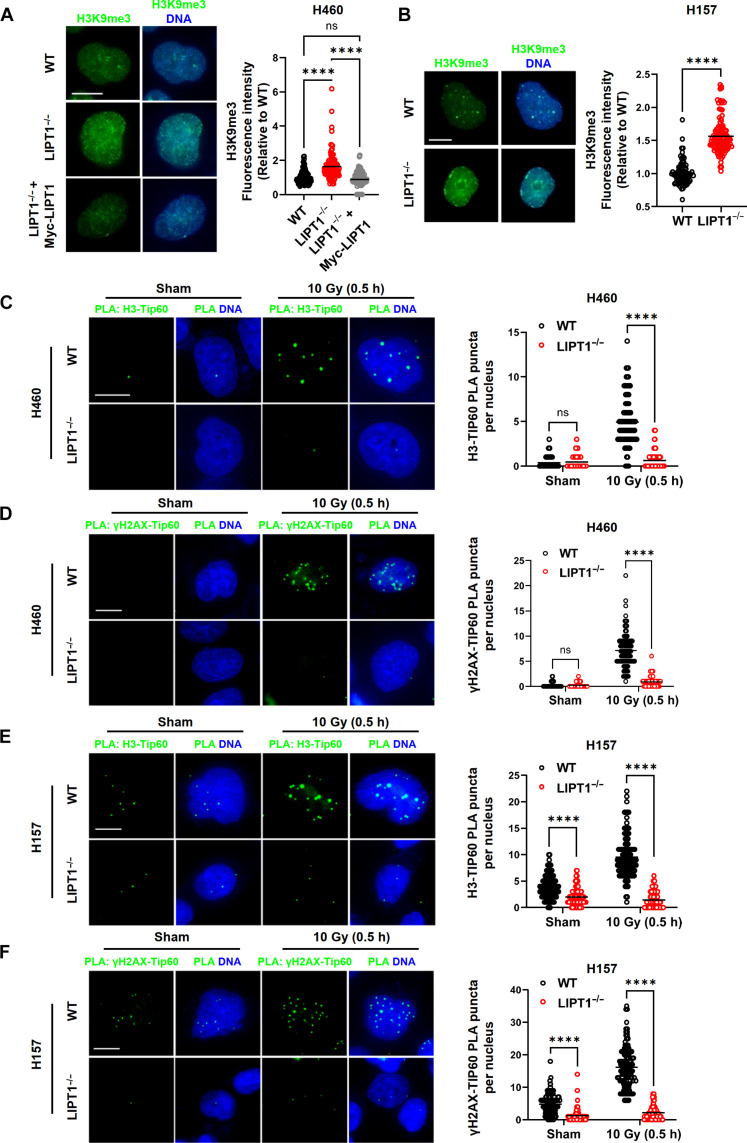
LIPT1^−/−^ cells exhibit elevated baseline H3K9me3 and impaired TIP60 recruitment to DNA damage sites. (**A**) Representative images and quantification of H3K9me3 by immunofluorescence staining in WT, LIPT1^−/−^ H460, and LIPT1^−/−^ H460 cells stably expressing Myc-LIPT1 cells. (**B**) Representative images and quantification of H3K9me3 by immunofluorescence staining in WT and LIPT1^−/−^ H157 cells. (**C** and **D**) Representative images and quantification of in situ proximity ligation assay (PLA, green dots) of interactions between histone H3 and TIP60 interaction (C) and between γH2AX and TIP60 (D) in WT and LIPT1^−/−^ H460 cells, with or without 10-Gy IR. (**E** and **F**) Representative images and quantification of in situ PLA (green dots) of interactions between histone H3 and TIP60 interaction (E) and between H2AX and TIP60 (F) in WT and LIPT1^−/−^ H157 cells with or without 10-Gy IR. For (A) to (F), nuclei were stained with Hoechst 33342. Scale bar, 10 μm. Imaging and quantification were performed on >100 cells per treatment. One-way ANOVA was used for the statistical analyses for (A), unpaired *t* test was used for (B), and two-way ANOVA was used for (C) to (F). ****P < 0.0001.

To test the hypothesis that elevated 2HG in LIPT1^−/−^ cells causes hypermethylation of H3K9 to impede the recruitment of TIP60, we first assessed the baseline levels of H3K9me3 via immunofluorescence staining in LIPT1^−/−^ cells. As expected, we observed elevated H3K9me3 at baseline in nuclei of LIPT1^−/−^ cells compared to WT cells; this was reversed by Myc-LIPT1 expression (Fig. 4, A and B).

To assess TIP60 recruitment, we performed proximity ligation assays (PLAs) to assess interactions between histone H3 and TIP60, as well as between γH2AX and TIP60. This revealed increased punctae for these interactions after 10-Gy IR in WT cells, indicative of TIP60 recruitment to DSB sites. However, these interactions were diminished in LIPT1^−/−^ cells, suggesting attenuated TIP60 recruitment (Fig. 4, C to F). These results support decreased TIP60 recruitment due to aberrant local H3K9me3 signaling observed in LIPT1^−/−^ cells.

### Depletion of LIPT1 impairs ATM-mediated DNA damage repair signaling cascade

**Fig. 5.**
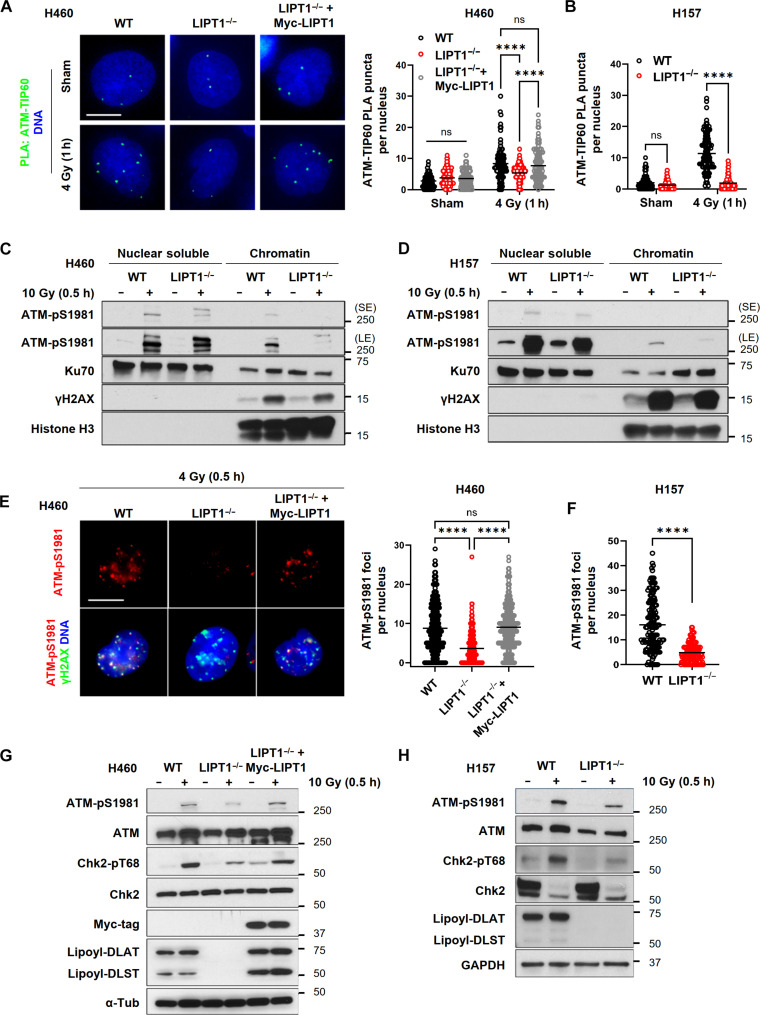
LIPT1^−/−^ cells have impaired activation of ATM and its downstream DNA damage repair signaling cascade. (**A**) Representative images and quantification of in situ PLA (green dots) of ATM and TIP60 interaction in nonirradiated control and 1 hour after 4 Gy in WT, LIPT1^−/−^ H460, and LIPT1^−/−^ H460 reconstituted Myc-LIPT1 cells. Nuclei were stained with Hoechst 33342. Scale bar, 10 μm. (**B**) Quantification of in situ PLA (green dots) of ATM and TIP60 interaction in nonirradiated control and 1 hour after 4 Gy in WT and LIPT1^−/−^ H157 cells. (**C** and **D**) Immunoblotting analysis of ATM-pS1981, Ku70, γH2AX, and histone 3 in the soluble nuclear and chromatin fractions of WT and LIPT1^−/−^ in H460 (C) and in H157 (D) cells, with or without IR (0.5 hours post 10 Gy). Histone H3 and γH2AX served as chromatin markers. LE, long exposure; SE, short exposure. (**E** and **F**) Immunofluorescence images and quantification of colocalized ATM-pS1981 (red) and γH2AX (green) foci at 0.5 hours post-4 Gy in WT, LIPT1^−/−^ H460, and Myc-LIPT1–reconstituted H460 cells (E), as well as WT and LIPT1^−/−^ H157 cells (F). Nuclei were stained with 4′,6-diamidino-2-phenylindole (DAPI). Scale bar, 10 μm. (**G** and **H**) Immunoblot analysis of ATM-pS1981, total ATM, Chk2-pT68, total Chk2, lipoyl-DLAT/DLST, and γH2AX in H460 (G) and H157 (H) cells at 0.5 hour post-10Gy. (A), (B), (E), and (F), quantification was performed on >100 cells per treatment. Two-way ANOVA was used for (A) and (B), one-way ANOVA was used for (E), and unpaired *t* test was used for (F). ****P < 0.0001.

Once recruited to the DSB sites, TIP60 acetylates ATM, promoting its activation by autophosphorylation at S1981 and initiating downstream signaling. To further evaluate the H3K9me3-TIP60-ATM–signaling axis, a PLA assay was conducted to assess the interaction between TIP60 and ATM at DSB sites. After exposure to 4-Gy IR, fewer foci indicative of decreased interactions between TIP60 and ATM were observed in the LIPT1^−/−^ cells compared to WT control, and this was rescued by stably expressing Myc-LIPT1(Fig. 5, A and B, and fig. S2A). In addition, acetylation of ATM was assessed through coimmunoprecipitation using a pan-acetyl-lysine antibody targeting ATM pull-down complexes. As expected, radiation induced ATM acetylation but to a lesser extent in the LIPT1^−/−^ cells (fig. S3).

To examine the activation of ATM in response to DNA DSBs, autophosphorylation of ATM at serine-1981 (S1981) was assessed by immunoblots in chromatin fractions and p-ATM foci formation by immunofluorescence staining. We used histone H3 and γH2AX as chromatin markers to ascertain the efficacy of subcellular fractionation of soluble nuclear lysates and insoluble chromatin fractions. We found that chromatin markers were present in the chromatin fraction but not in the soluble fraction. Immunoblot analysis showed a decrease in radiation-induced ATM phosphorylation (p-S1981) in the chromatin-fractions of LIPT1^−/−^ cells compared to the WT cells (Fig. 5, C and D), suggestive of attenuated activation of ATM. Assessment of p-ATM foci formation in response to radiation via immunofluorescence further corroborated the attenuation of p-ATM activation within the nuclei of LIPT1^−/−^ cells, which was restored by Myc-LIPT1 expression (Fig. 5, E and F, and fig. S2B).

Downstream DNA damage response signaling was also attenuated in LIPT1^−/−^ cells, as evidenced by diminished phosphorylation of the ATM effector CHK2 (Fig. 5, G and H). Collectively, these findings underscore the key role of LIPT1 in modulating the H3K9me3-TIP60-ATM activation and downstream signaling for DNA damage repair.

### LIPT1^−/−^ cells are functionally deficient in HR repair

Homologous recombination (HR) ensures precise repair of DSBs by using undamaged sister chromatids as templates (*44*, *45*). TIP60 and ATM are two key proximal HR repair proteins that, once activated, further recruit and phosphorylate downstream HR components such as EXO1, RPA, BRCA2, and RAD51 (*44*, *46*).

**Fig. 6.**
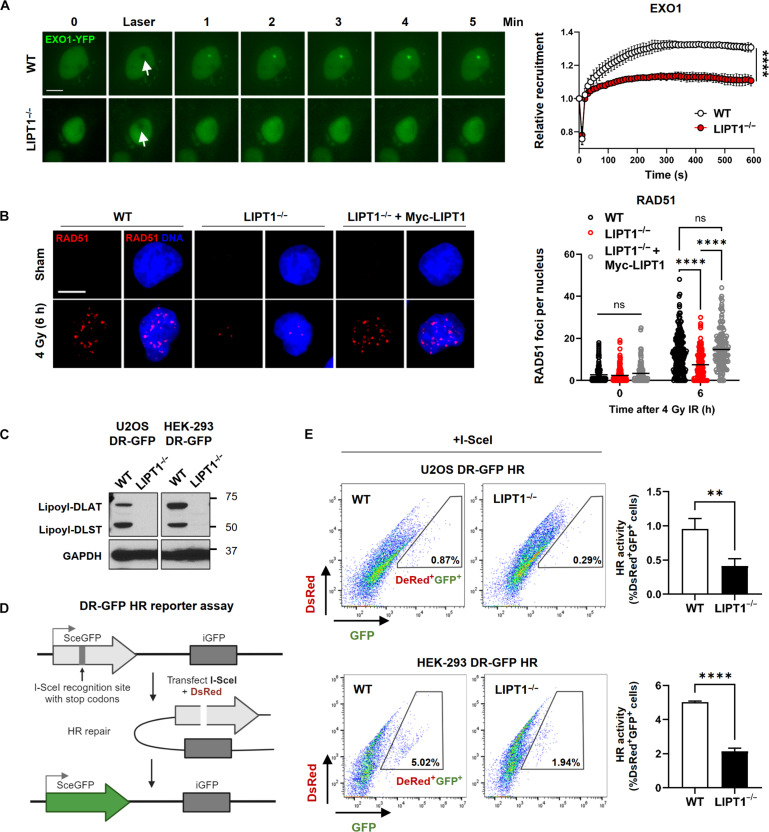
LIPT1^−/−^ cells are functionally deficient in HR repair. (**A**) Recruitment of EXO1–yellow fluorescent protein to laser-generated DSBs in single living WT and LIPT1^−/−^ H157 cells. Relative fluorescence intensity of the local accumulation of EXO1 at laser-induced DNA damage sites was quantified for over 10 min and displayed as mean ± SD in WT and LIPT1^−/−^ H157 cells. Simple linear regression was used for the statistical analyses. ****P < 0.0001. Scale bar, 10 μm. (**B**) Representative images and quantification of RAD51 foci by immunofluorescence staining in nonirradiated control and at 6 hours after 4 Gy in WT, LIPT1^−/−^ H460, and LIPT1^−/−^ H460 stably expressing Myc-LIPT1 cells. Nuclei were stained with DAPI. Scale bar, 10 μm. Imaging and quantification were performed on >100 cells per treatment. Two-way ANOVA was used for the statistical analyses. ****P < 0.0001. (**C**) Representative immunoblots demonstrate knockout of LIPT1 in U2OS and HEK-293 DR-GFP HR cells using the CRISPR-Cas9 gene editing system. Lipoylation was detected on DLAT and DLST, with GAPDH serving as the internal control. (**D**) Schematic illustration of the DR-GFP HR reporter assay. (**E**) HR activity assay by quantifying the population of cells positive for both DsRed and GFP in WT and LIPT1^−/−^ U2OS (top) and WT and LIPT1^−/−^ HEK-293 DR-GFP HR cells (bottom). Data were represented as means ± SD, unpaired *t* tests were used for the statistical analyses. **P < 0.01, ****P < 0.0001.

To investigate the activation of HR repair proteins downstream of ATM, we tracked the recruitment of green fluorescent protein (GFP)–tagged EXO1 in response to laser-induced DSBs. Exonuclease 1 (EXO1) is instrumental in DNA end resection during HR repair, generating single-stranded DNA (ssDNA) overhangs for subsequent repair steps (*47*). Within seconds to 1 min after damage, we found EXO1 localized to the damage site in the WT cells; however, its recruitment was attenuated in LIPT1^−/−^ cells (Fig. 6A). Furthermore, the recruitment and activation of recombinase RAD51, which is responsible for loading onto ssDNA to form a nucleoprotein filament facilitating homology search and strand exchange with intact sister chromatids (*48*), was also diminished after IR in LIPT1^−/−^ cells. This was evidenced by decreased RAD51 foci numbers after radiation in LIPT1^−/−^ cells compared to the control, which were restored by Myc-LIPT1 expression (Fig. 6B).

To directly measure HR repair efficiency, we obtained DR-GFP HR reporter cell systems established in human embryonic kidney (HEK) 293 and U2OS cells and generated LIPT1^−/−^ and nontargeting control WT cells using CRISPR editing (Fig. 6C). The reporter cell lines contain an inactive expression cassette for GFP interrupted by recognition sites for endonuclease I-SceI. Transient transfection of I-SceI (dsRed) leads to DSBs, and HR repair restores GFP expression, which is quantified by fluorescence-activated cell sorting (FACS) analysis (Fig. 6D) (*49*). LIPT1 deficiency resulted in a decrease in cells positive for both DsRed and GFP, indicating decreased HR repair efficiency in HEK293 and U2OS cells (Fig. 6E).

### 2HG inhibition of KDM4B drives the DNA damage phenotype in LIPT1^−/−^ cells

**Fig. 7.**
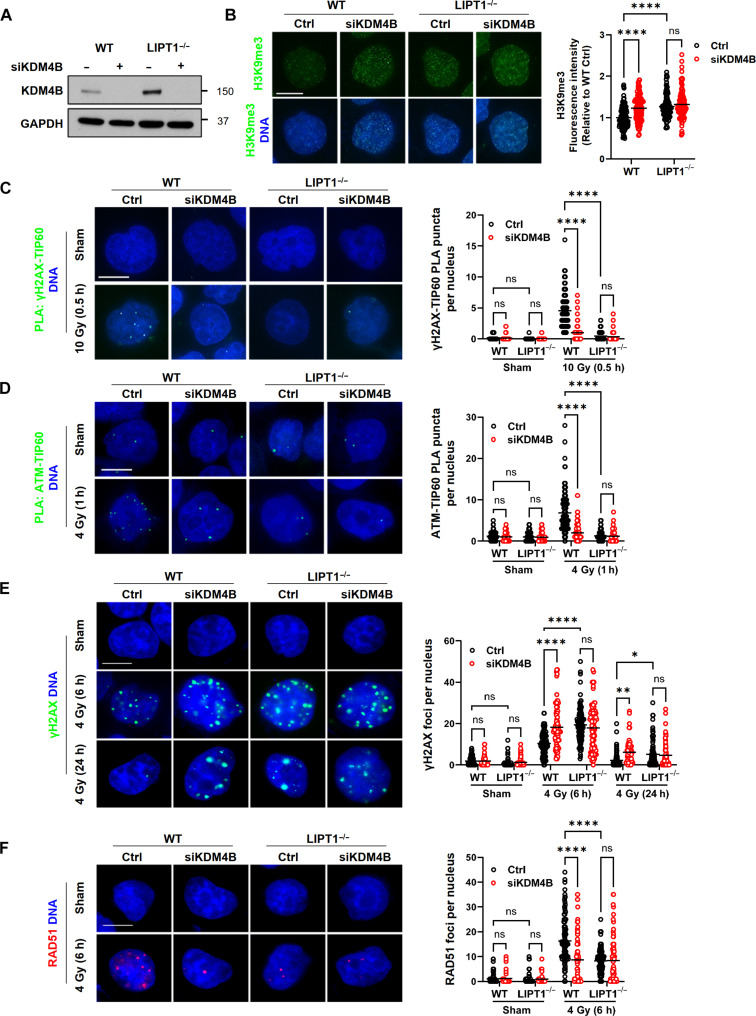
Inhibition of KDM4B disrupts the H3K9me3-TIP60-ATM signaling axis and HR repair. (**A**) Representative immunoblots validating siRNA suppression of KDM4B in WT and LIPT1^−/−^ H460 cells, with GAPDH used as a loading control. (**B**) Representative images and quantification of H3K9me3 by immunofluorescence staining in Ctrl and siKDM4B WT and LIPT1^−/−^ H460 cells. (**C** and **D**) Representative images and quantification of in situ PLA (green dots) of interactions between γH2AX and TIP60 interaction (C), and between ATM and TIP60 (D) in Ctrl and siKDM4B WT and LIPT1^−/−^ H460 cells, with or without 10- and 4-Gy IR. (**E**) Representative images and quantification of γH2AX foci by immunofluorescence staining in nonirradiated cells and at 6 and 24 hours after 4-Gy IR in Ctrl and siKDM4B WT and LIPT1^−/−^ H460 cells. (**F**) Representative images and quantification of RAD51 foci by immunofluorescence staining in nonirradiated cells and 6 hours after 4-Gy IR in Ctrl and siKDM4B WT and LIPT1^−/−^ H460 cells. For (B) to (F), nuclei were stained with Hoechst 33342. Scale bar, 10 μm. Imaging and quantification were performed on >100 cells per treatment. Two-way ANOVA was used for the statistical analyses. *P < 0.05, **P < 0.01, ****P < 0.0001.

The histone demethylase KDM4B belongs to a family of α-KG–dependent dioxygenases and regulates levels of H3K9me3. KDM4B inhibition by 2HG impairs HR repair in isocitrate dehydrogenase (IDH) mutant gliomas (*33*). To determine the contribution of 2HG-mediated KDM4B inhibition to the HR deficiency and radiosensitivity observed in LIPT1^−/−^ cells, we silenced KDM4B in WT cells to recapitulate the LIPT1^−/−^ phenotype and conducted rescue experiments through 2HG depletion or competition in LIPT1^−/−^ cells. First, we confirmed KDM4B knockdown in both WT and LIPT1^−/−^ H460 by immunoblotting (Fig. 7A). Baseline KDM4B expression was elevated in LIPT1^−/−^ cells compared to WT, perhaps reflecting a compensatory response to reduced enzymatic activity. Knocking down KDM4B in WT cells increased baseline H3K9me3 levels to those observed in LIPT1^−/−^ cells, whereas KDM4B knockdown in LIPT1^−/−^ cells did not further elevate H3K9me3 levels (Fig. 7B). KDM4B knockdown in WT cells also recapitulated the DNA damage phenotype of LIPT1^−/−^ cells, including decreased interactions between γH2A and TIP60 and between TIP60 and ATM, as shown by PLA (Fig. 7, C and D). Following radiation exposure, KDM4B knockdown in WT cells increased γH2AX and reduced RAD51 recruitment to levels comparable to LIPT1^−/−^ cells (Fig. 7, E and F).

2HG also inhibits other α-KG–dependent demethylases, as evidenced by increased H3K4me3 and H3K27me3 in LIPT1^−/−^ cells (fig. S4). Although we did not completely delineate the contribution of other α-KG–dependent demethylases besides KDM4B, we observed that silencing a close subfamily member, KDM4A, did not alter H3K9me3 or RAD51 levels (fig. S5). This result is consistent with a prior report that cells rely on KDM4B for H3k9me3 demethylation more than KDM4A (*33*). Collectively, these results indicate that KDM4B is a main driver of the DNA damage phenotype in LIPT1^−/−^ cells.

**Fig. 8.**
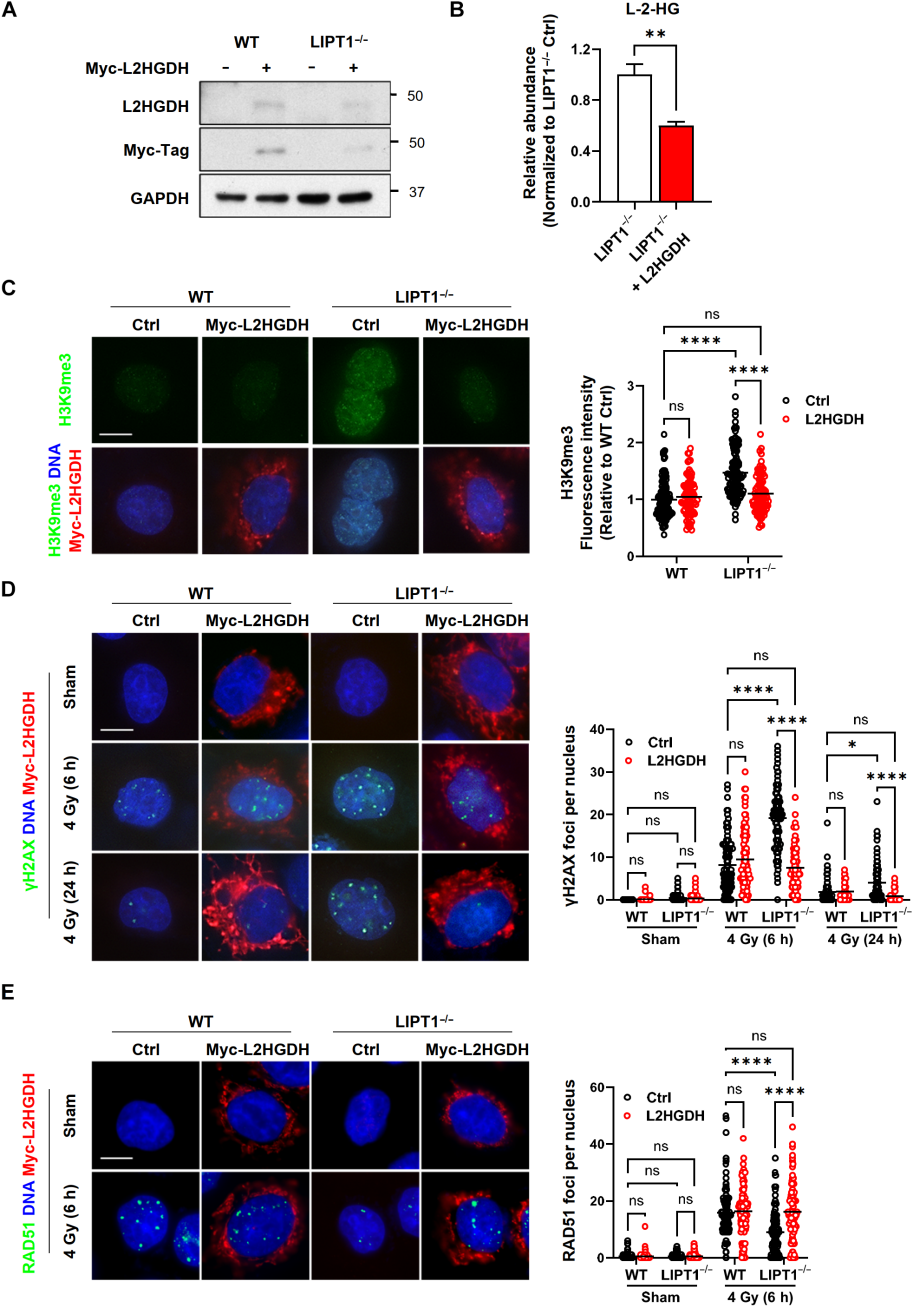
L2HGDH overexpression normalizes H3K9me3 and reverses DNA damage phenotypes in LIPT1^−/−^ H460 cells. (**A**) Representative immunoblots of Myc-L2HGDH overexpression in WT and LIPT1^−/−^ H460 cells, with GAPDH used as a loading control. (**B**) Relative abundance of L-2-hydroxyglutarate (2HG) in nontransfected control cells and flow sorted GPF-L2HGDH–positive LIPT1^−/−^ H460 cells. Data were represented as mean ± SD, and unpaired *t* tests were used for the statistical analyses. **P < 0.01. (**C**) Representative images and quantification of H3K9me3 by immunofluorescence staining in nontransfected control and Myc-L2HGDH–positive WT and LIPT1^−/−^ H460 cells. (**D**) Representative images and quantification of γH2AX foci by immunofluorescence staining in nonirradiated cells and at 6 and 24 hours after 4-Gy IR in Ctrl and Myc-L2HGDH–positive WT and LIPT1^−/−^ H460 cells. (**E**) Representative images and quantification of RAD51 foci by immunofluorescence staining in nonirradiated control and at 6 hours after 4 Gy in Ctrl and Myc-L2HGDH–positive WT and LIPT1^−/−^ H460 cells. For (C) to (E), nuclei were stained with Hoechst 33342. Scale bar, 10 μm. Imaging and quantification were performed on >80 to 100 cells per treatment. Two-way ANOVA was used for the statistical analyses. *P < 0.05, ****P < 0.0001.

Next, we transiently expressed GPF-tagged L-2-hydroxyglutarate dehydrogenase (L2HGDH), the enzyme responsible for metabolizing L-2HG to α-KG, in LIPT1^−/−^ cells (Fig. 8A). As expected, L2HGDH overexpression reduced L-2HG levels in LIPT1^−/−^ H460 cells (Fig. 8B). This intervention also reversed key aspects of the DNA damage phenotype associated with LIPT1 loss, including normalization of H3K9me3 levels (Fig. 8C), reduction of γH2AX (Fig. 8D) and restoration of RAD51 recruitment following IR (Fig. 8E).

**Fig. 9.**
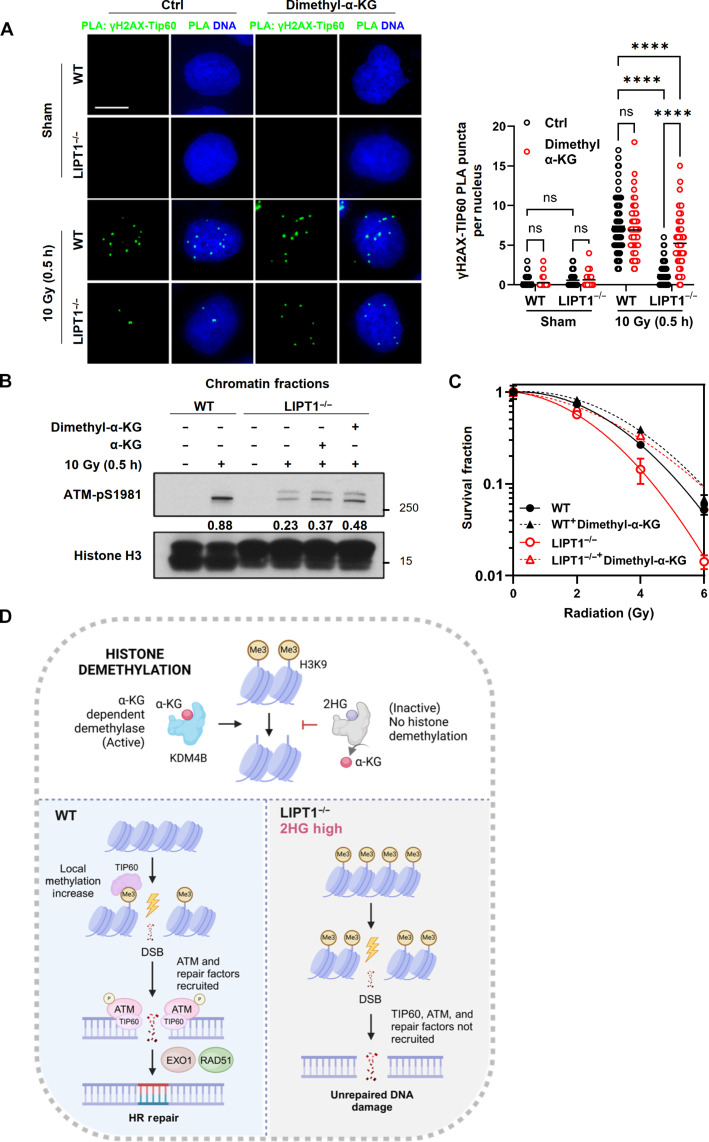
α-KG supplementation restores DNA damage repair and enhances survival of LIPT1^−/−^ H460 cells after radiation. (**A**) Representative images and quantification of in situ PLA (green dots) of interactions between γH2AX and TIP60 in WT and LIPT1^−/−^ H460 cells with or without 1 mM dimethyl–α-KG and 10-Gy IR. Nuclei were stained with Hoechst 33342. Scale bar, 10 μm. Imaging and quantification were performed on >100 cells per treatment. Two-way ANOVA was used for the statistical analyses. ****P < 0.0001. (**B**) Immunoblotting analysis of ATM-pS1981 and histone H3 in the chromatin fractions of WT and LIPT1^−/−^ in H460 cells with or without 1 mM dimethyl–α-KG, 1 mM α-KG, and 10-Gy IR. Histone H3 was used as marker and internal control for the chromatin fraction. Protein levels of ATM-pS1981 were normalized with internal control histone H3. (**C**) Clonogenic assay of WT and LIPT1^−/−^ H460 cells with or without 1 mM dimethyl–α-KG after 2, 4, and 6 Gy. The surviving fraction was normalized to the corresponding sham control and survival curves were fitted using the linear-quadratic model. (**D**) Schematic illustrating how LIPT1 deficiency impairs the IR-induced, TIP60-ATM–mediated HR damage repair pathway due to a deficiency in α-KG–dependent demethylation.

To further test whether exogenous α-KG could outcompete 2HG in restoring DNA repair and survival after radiation, we treated WT and LIPT1^−/−^ H460 cells with 1 mM dimethyl-α-KG for 24 hours before radiation and maintained the treatment until completion of the assays. This condition is comparable to prior work demonstrating that α-KG can outcompete 2HG and restore KDM4B function (*33*). We found that exogenous α-KG partially but significantly restored the interaction between ATM and TIP60 in LIPT1^−/−^ H460 cells after radiation, as shown by PLA (Fig. 9A). Immunoblot analysis of the chromatin fractions also revealed a partial rescue of ATM phosphorylation (pS1981) by exogenous α-KG (Fig. 9B). Moreover, supplementation with either dimethyl–α-KG or α-KG rescued the survival of LIPT1^−/−^ H460 cells after various doses of radiation to levels comparable to WT H460 cells (Fig. 9C and fig. S6A). In contrast, supplementation of malate, another TCA cycle intermediate, did not rescue radiosensitivity conferred by LIPT1 knockout (fig. S6B). In summary, our data elucidate a key function of LIPT1 in sustaining the H3K9me3-TIP60-ATM signaling axis to recruit HR repair components in response to DNA DSB damage (Fig. 9D). Specifically, LIPT1 deficiency disrupts the lipoylation and function of PDH and α-KGDH within the TCA cycle, leading to metabolic perturbations, particularly the accumulation of 2HG. This accumulation impedes KDM4B and disrupts the H3K9me3-TIP60-ATM signaling axis. Consequently, key HR repair components downstream of ATM, such as EXO1 and RAD51, exhibit impaired recruitment to DNA damage sites, resulting in decreased HR repair efficiency and the accumulation of unrepaired DNA damage.

### Inhibition of lipoylation enhances NSCLC’s radiation response in vivo

**Fig. 10.**
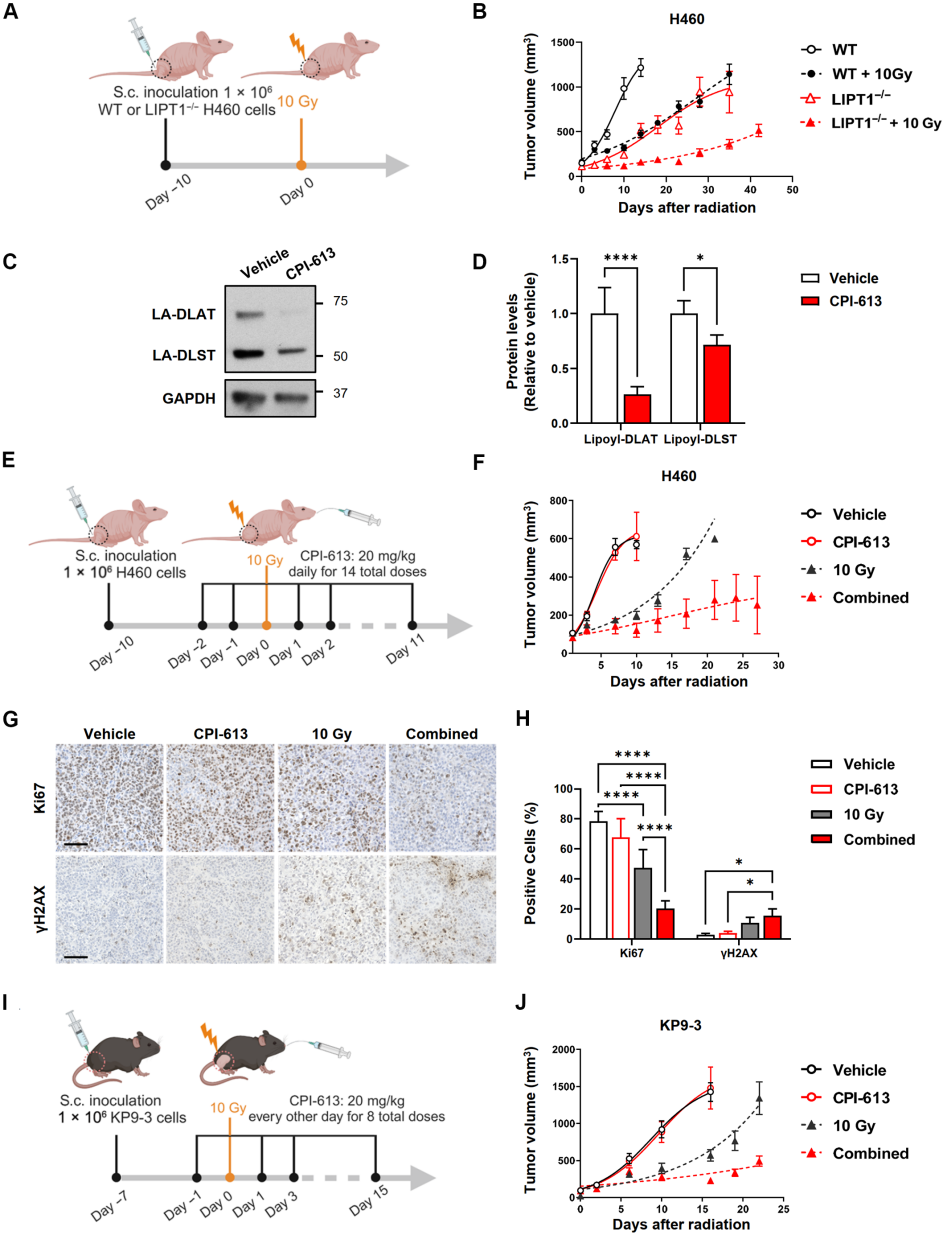
Inhibition of lipoylation enhances NSCLC’s radiation response in vivo. (**A**) Schematic illustrating timeline of WT and LIPT1^−/−^ H460 tumor inoculation and treatments. (**B**) Tumor growth rate of WT and LIPT1^−/−^ H460 xenografts in athymic nude mice with or without 10 Gy IR. *n* = 8 to 10 tumors. (**C** and **D**) Representative images (C) and quantification (D) of immunoblotting analysis of lipoylated-DLAT and DLST using pooled tumors from H460 tumor–bearing athymic nude mice that either received vehicle or CPI-613 (20 mg/kg) for 14 doses. Intensity was quantified by ImageJ and normalized by GAPDH, *n* = 4. Data were represented as means ± SD, and two-way ANOVA was used for the statistical analyses. *P < 0.05, ****P < 0.0001. (**E**) Schematic illustrating timeline of tumor inoculation and treatments. Mice received daily treatment with either CPI-613 (20 mg/kg) or vehicle daily for 14 days. In the irradiated group, 10-Gy IR was administered to tumors after the first two doses of CPI-613. (**F**) Tumor growth rate of WT H460 xenografts in athymic nude mice treated with vehicle, CPI-613, 10-Gy, or combined treatment. *n* = 7 to 10 tumors. (**G** and **H**) Representative images (G) and quantification (H) of Ki67 and γH2AX-positive nuclei by immunohistochemistry staining. Scale bar, 100 μm. Data were represented as means ± SD, and two-way ANOVA was used for the statistical analyses. *P < 0.05, ****P < 0.0001. (**I**) Schematic illustrating timeline of KP9-3 tumor inoculation and treatments. C57B/L6 mice received daily treatment with either CPI-613 (20 mg/kg) or vehicle every other day for a total of eight doses. In irradiated group, 10-Gy IR was administered to tumor between the first two doses of CPI-613. (**J**) Tumor growth rate of KP9-3 xenografts in C57B/L6 mice treated with vehicle, CPI-613, 10-Gy, or combined treatment. *n* = 8 to 12 mice. For (A), (C), and (G), error bars represent the SEM.

To test whether LIPT1 deficiency confers radiosensitivity in vivo, we subcutaneously inoculated WT and LIPT1^−/−^ H460 cells into the flanks of athymic nude mice. Once tumors reached a palpable size, a single 10-Gy dose of radiation was administered to the tumor site, and tumor growth was monitored (Fig. 10A). We found that LIPT1^−/−^ tumors grew more slowly compared to the control. Following 10-Gy IR, the tumor growth delay was significantly greater in LIPT1^−/−^ tumors compared to the control tumors (Fig. 10B).

Next, we performed pharmacological validation using CPI-613 (Devimistat), an inhibitory analog of LA that binds to E2 subunits, disrupting the enzymatic function of the PDH and α-KGDH complexes (*50*). We first confirmed by immunoblot that CPI-613 decreases lipoylated-DLAT and lipoylated-DLST levels both in vitro and in vivo (Fig. 10, C and D, and fig. S7A). We then conducted metabolomics analysis on H460 cells treated with vehicle or CPI-613 at 50 and 100 μM for 24 hours. Consistent with LIPT1^−/−^ H460 cells (Fig. 3), CPI-613 reduced the abundance of TCA cycle intermediates and led to perturbation of expected pathways including the TCA cycle; alanine, aspartate and glutamate metabolism; arginine and proline; and nucleotide metabolism (fig. S7, B to E). Furthermore, like LIPT1^−/−^ cells, CPI-613–treated cells demonstrated increased 2HG and baseline H3K9 trimethylation and decreased the survival and γH2AX foci resolution after IR, indicating impaired DNA damage repair (fig. S7, F to I). CPI-613 treatment in HeLa cells produced similar results (fig. S8).

We orally administered vehicle or CPI-613 to H460 tumor–bearing athymic nude mice with or without one fraction of 10-Gy radiation to tumors (Fig. 10E). We found that CPI-613 alone did not decrease tumor growth but enhanced the effect of radiation to reduce tumor growth (Fig. 10F). Immunostaining of the proliferation marker Ki67 in tumor sections showed reduced cell proliferation in the radiated tumors, with an even more pronounced reduction in tumors receiving combination treatment. The combination treatment also enhanced expression of γH2AX over untreated tumors and tumors treated with CPI-613 alone (Fig. 10, G and H).

We next used KP9-3, a C57B/L6-derived murine lung adenocarcinoma cell line driven by oncogenic *Kras* and *Tp53* loss, to test the radiosensitization strategy in a syngeneic model (*51*). We administered CPI-613 once every other day for a total of eight doses (Fig. 10I) and found that the drug enhanced the efficacy of radiation therapy in this immunocompetent model (Fig. 10J). Thus, lipoylation inhibition enhances radiation responses in multiple in vivo models of NSCLC using both pharmacological and genetic approaches.

## DISCUSSION

Altered metabolism is an actionable hallmark of cancer (*52*). However, the optimal way to leverage metabolic pathways to enhance the outcomes of radiation therapy in lung cancer remains unknown. CRISPR-Cas9 has emerged as a powerful genetic screening tool in cancer research. Compared to small interfering RNA (siRNA) screen, CRISPR-Cas9 offers the advantages of complete gene knockout, fewer off-target effects, and improved reproducibility, making it uniquely suited for dissecting complex cellular processes, such as metabolism, and for identifying therapeutic vulnerabilities. Recent whole-genome CRISPR-Cas9 screens have identified various drivers of radioresistance in different cancer cell lines. For example, stimulator of interferon genes (STING) was identified as the sole statistically significant target in a screen conducted in head and neck squamous cell carcinoma (*53*), while LUC7L2 was found to drive radioresistance in nasopharyngeal cancer by up-regulating autophagy (*54*). In brain tumors, CARHSP1 was found to drive radioresistance by up-regulating inflammation signaling (*55*).

Our CRISPR screen for metabolic vulnerabilities during ionizing radiation identified genes involved in redox balance and DNA damage repair, aligning with the known biological functions of ionizing radiation and previous siRNA screens using radiation (*56*). Unexpectedly, we uncovered lipoylation as a metabolic vulnerability in gamma-irradiated cells. Our results revealed a key function of LIPT1 in regulating high-fidelity homology-based repair and maintaining chromosome stability. This function is at least partially explained by the inhibition of KDM4B by 2HG, impairing the recruitment of DNA damage repair machinery and signaling cascades. It is possible that the increased DNA damage associated with LIPT1 loss may be attributed to other histone demethylases, such as KDM5B, that are known to modulate radiation sensitivity (*57*), as well as additional aspects of DNA repair, such as NHEJ and replication stress. Notably, among the top 10 targets, HIF1AN and PHF8 belong to the Jumonji domain–containing protein family known as methylation-dependent histone modifiers (*58*). PHF8 functions as 2-ketoglutarate–dependent histone demethylase for H3K9me1/2, and HIF1AN directly regulates 2-ketoglutarate–dependent dioxygenases (*59*). These findings highlight the essential role of histone methylation in radiation response regulated by TCA cycle oncometabolites.

Collectively, these results support targeting lipoylation as a potential strategy to improve radiation outcomes in diligently selected patients with cancer. First, metabolic compensation and plasticity can result in resistance to metabolic therapies. Compared to targeting a single enzyme, targeting lipoylation inhibits the TCA cycle at multiple entry points, potentially making therapeutic effects more achievable and less prone to therapy evasion. The TCA cycle’s contributions to ATP, NAD^+^, NADPH, and de novo nucleotide synthesis may also contribute to radiosensitization under conditions of lipoylation suppression. Second, isotope tracing studies conducted in patients provide evidence that NSCLCs oxidize pyruvate derived from glucose and lactate in the TCA cycle (*60*, *61*). Glucose’s contribution to the TCA cycle is higher in human NSCLCs than in surrounding lung, suggesting that targeting lipoylation may preferentially radiosensitize tumors. Third, clinical trials found CPI-613 to be generally well tolerated with few severe side effects in patients with cancer, raising the possibility that it could be safely combined with radiation or other therapies that induce DNA damage.

Although our work focused on NSCLC, lipoylation inhibition as a radiosensitization strategy may be applicable to other primary cancer types beyond lung cancer. We demonstrated that LIPT1 knockout leads to HR repair defect in established osteosarcoma cell line (U2OS) and HEK-293 HR reporter cell lines (Fig. 6, C to E). Recently, CPI-613 has been found to radiosensitize pancreatic cancer cells in vitro (*62*), and a phase 1 clinical study combining CPI613 and radiation in pancreatic cancer (NCT05325281) has been initiated.

Successful clinic translation depends on matching metabolic therapies with tumor metabolism and using predictive biomarkers. Metabolic studies in patients with cancer indicate that tumors originating from different primary sites differ in their ability to carry out glucose oxidation (*60*, *61*, *63*). We postulate that tumors with robust TCA cycle activity would be well-suited for this approach, as this is predicted to enhance drug sensitivity. In contrast, primary clear cell renal cell carcinomas exhibit depressed glucose oxidation (*63*) and may be less well-suited to this strategy. The lipoylation state of 2-ketoacid dehydrogenases, *LIPT1* expression and 2HG abundance may serve as predictive biomarkers to guide therapeutic interventions. Recent analyses based on cuproptosis-related genes have linked *LIPT1* with cancer outcomes. Studies in hepatocellular carcinoma (*64*, *65*) and acute myeloid leukemia (AML) (*66*) suggest that *LIPT1* expression is elevated and associated with poor prognosis, whereas another study in lung cancer found the opposite (*67*). This perhaps reflects LIPT1’s role in promoting growth and proliferation and its antitumor properties by supporting cuproptosis and homology-based repair. Comprehensive studies to thoroughly assess the efficacy and toxicity of lipoylation suppression combined with radiation in additional preclinical models will be essential to determine the clinical and translational potential of the findings. In addition, mutations in *IDH1/2* genes, commonly occurring in glioma and AML and less frequently in other tumors, lead to elevated 2HG. On the basis of our findings and those from other studies (*32*, *68*), we postulate that tumors with low/absent 2HG and high LIPT1 expression may respond well to lipoylation inhibition, whereas tumors with high 2HG and low LIPT1 may respond well to DNA-damaging agents. Preclinical studies and clinical trials are being pursued to explore a personalized, metabolism-guided, radiosensitization strategy.

The link between lipoylation, DNA damage repair, and chromosome stability has clinical relevance beyond cancer. Mutations in LIPT1, LIAS, and DLAT have been reported in human, along with hundreds of cases involving mutations in 2-ketoacid dehydrogenase (*12*). It is unknown whether 2HG elevations in these patients can lead to clinically relevant, aberrant histone remodeling, predisposing patients to cancer and heightened sensitivity to environmental radiation, ultraviolet, and DNA-damaging agents. Future studies in mice and patients are needed. In addition, protein lipoylation plays a critical role in mitochondrial health, which has been linked to normal and disease processes including aging, obesity, and diabetes. Our findings further shed light on the pathogenesis and treatment of these benign conditions.

## MATERIALS AND METHODS

### Cell and cell culture

The human NSCLC lines H460 and H157 and murine lung cancer cell line KP9-3 were provided by J. Minna (Hamon Center Collection at UT Southwestern Medical Center, TX, USA) and maintained in RPMI 1640 medium (HyClone, Cytiva, Marlborough, MA) supplemented with 5% fetal bovine serum (FBS, Gemini Bio-Products, West Sacramento, CA), 2 mM glutamine (Thermo Fisher Scientific, Waltham, MA), and 1% penicillin-streptomycin (P/S) (Gibco, Thermo Fisher Scientific). HEK293T cells (CRL-3216) used for lentiviral production and HeLa cells (CCL-2) were obtained from the American Type Culture Collection and cultured in Dulbecco’s modified Eagle’s medium (DMEM) supplemented with 10% FBS and 1% P/S. The DR-GFP U2OS and HEK293 cell lines (*49*) were gifts from J. M. Stark (Beckman Research Institute of the City of Hope, CA, USA) and B. Chen (UT Southwestern Medical Center, TX, USA) and cultured in DMEM with 10% FBS and 1% P/S. All cells were cultured in a humidified incubator with 5% CO_2_ at 37°C and were used within 15 passages after resuscitation from the original stocks were used. Routine testing using a mycoplasma PCR detection kit (Bulldog Bio, Portsmouth, NH) was performed to ensure absence of mycoplasma cell culture contamination. All cell lines were authenticated by DNA fingerprinting and tested negative for mycoplasma.

### Design and cloning of sgRNAs

To establish the CRISPR-Cas9 LIPT1 knockout cell lines, gRNAs were selected using the GenCRISPR gRNA design tool (https://genscript.com/tools/gRNA-design-tool). The gRNA sequences used were as follows: *LIPT1* sgRNA sequence (TGG TAG CCT GCA CAT CCA GC) and nontargeting gRNA control sequence (TTC TTA GAA GTT GCT CCA C). The gRNA oligonucleotide duplexes were cloned into the pSpCas9(BB)-2A-GFP (PX458) (Addgene, plasmid # 48138) and lentiCRISPR v2-Blast (Addgene, plasmid #52962) vectors, following the published protocol (*69*, *70*). Briefly, guide oligonucleotides were synthesized and annealed in the following reaction: 10 μM of each sense and antisense oligonucleotide, T4 ligation buffer (1×), and 5 U of T4 polynucleotide kinase (New England Biolabs, Ipswich, MA) with the cycling parameters of 37°C for 30 min, 95°C for 5 min, and then ramp down to 25°C at 5°C/min. The annealed oligonucleotides were cloned into the sgRNA vectors using a Golden Gate assembly strategy including: 100 ng of circular sgRNA vector plasmid, 0.2 μM of annealed oligonucleotides, NEBuffer 2 (1×); 20 U of BbsI or BsmBI, 0.2 mM of ATP, bovine serum albumin (BSA, 0.1 mg ml^−1^), and 750 U of T4 DNA ligase (New England Biolabs) with the cycling parameters of 20 cycles at 37°C for 5 min, 20°C for 5 min, followed by 80°C incubation for 20 min.

### Generation of LIPT1^−/−^ and Myc-LIPT1 cell lines

H460 cells were transfected with control and *LIPT1* gRNA-pX458 plasmids using Lipofectamine 3000 transfection reagent (Thermo Fisher Scientific) and sorted by the BD FACSAria III Cell Sorter (BD biosciences, Franklin Lakes, NJ) based on GFP expression to isolate single cells. Lentiviruses containing LIPT1 targeting or control sgRNAs were packaged in HEK293T cells. Specifically, 2 μg of psPAX2 (Addgene, #12260), 1 μg of pMD2.G (Addgene, #12259), and 5 μg of sgRNA vectors were transfected into HEK293T cells seeded in each 10-cm petri dish. Lentiviruses were collected by collecting and filtering the supernatant 48 to 72 hours posttransfection. H157 and DR-GFP cells were transduced with *LIPT1* gRNA-lentiCRISPR v2 viral particles generated from transfected HEK293T cells, selected with blasticidin (10 μg/ml) and separated into single cells. The LIPT1 knockout cells were cultured in medium with uridine (100 μg/ml; MilliporeSigma, Burlington, MA) and 1 mM sodium pyruvate (MilliporeSigma) and confirmed by assessing the expression levels of lipoylation on DLAT and DLST protein in single cell–derived clones. To reexpress Myc-tagged LIPT1 in LIPT1^−/−^ cells, LIPT1^−/−^ H460 cells were transduced with pLenti-EF1a-C-Myc-DDK-IRES-Puro-LIPT1-WT (*13*) lentivirus generated in HEK293T and selected with puromycin (1 μg/ml). The expression of Myc-LIPT1 was confirmed by Myc-tag antibody.

### CRISPR screen

The human CRISPR knockout pooled library was designed to target 2981 metabolic genes (Addgene, #110066) (*71*) and synthesized as a pool of lentiviral vectors containing unique gRNA sequences (*70*). Eight individual sgRNAs were designed for each gene, and 1000 negative control nontargeting sgRNAs were selected from the GeCKO CRISPR libraries (*72*) totaling 24,848 sgRNA. The sgRNA library was purified and prepared, and lentiviruses were produced as previously described (*73*, *74*). To generate an NSCLC line for CRISPR-based loss-of-function screens, H157 cells were transduced with the lentiviral CRISPR library, ensuring a low appropriate MOI of approximately 0.24 to facilitate sgRNA integration into the cellular genome. Two days after transduction, the cells underwent puromycin (1 μg/ml) selection for 5 days and then were cultured in fresh medium without puromycin for 24 hours. Five individual flasks of at least 25 million cells (>1000×) were irradiated with 10 Gy using a Mark-II Cesium-137 irradiator (JL Shepherd and Associates, San Fernando, CA), with five additional unirradiated flasks serving as controls within the same experiment. Each flask was cultured and maintained between 25 and 125 million total cells to maintain a library coverage of at least 1000 cells per sgRNA at all times. Genomic DNA was then extracted from all samples at day 14, followed by amplification of the integrated gRNA sequences via two-step PCR (*75*), maintaining that approximately 1000× coverage of the library Amplicon sequencing was performed on an Illumina NextSeq 500 sequencer. sgRNA sequences were extracted from FASTQ files and aligned to the sgRNA sequences of the CRISPR screen library. The reads of each sgRNA were counted and normalized to the total read counts for each sample. Pearson correlations between replicates were calculated using the log_2_-transformed sgRNA counts. The counts of each sgRNA were then counted from the sgRNA sequences with the MAGeCK (v.0.5.5) “count” function (*76*). The statistical significance of individual gRNAs was determined by leveraging the learned mean-variance model, with robust rank aggregation used to identify essential or enriched genes (*73*). A significance threshold of false discovery rate–corrected *P* value ≤0.05 was applied to delineate genes of statistical significance. Details for CRISPR library sgRNA sequences, PCR primers, raw sgRNA guide counts, statistics, and ranking of genes comparing irradiated samples and controls are listed in table S1.

### In vitro radiation and drug treatments

For ionizing radiation (IR) treatment, cells were exposed to 2- to 10-Gy IR using a Mark-II Cesium-137 irradiator (JL Shepherd and Associates, San Fernando, CA). For pharmacological treatment, cells were treated with CPI-613 (ApexBio, Houston, TX) dissolved in dimethyl sulfoxide (DMSO) at the indicated concentrations. For α-KG rescue experiments, cells were pretreated with 1 mM dimethyl- α-KG or α-KG (MilliporeSigma) 24 hours before radiation and indicated assays.

### Plasmid and siRNA transfection

For L2HGDH overexpression, H460 WT and LIPT1^−/−^ cells were transfected with Myc-DDK–tagged L2HGDH or tGFP-tagged L2HGDH plasmids (RG217631 and RC217631, OriGene) using Lipofectamine 3000 transfection reagent (Thermo Fisher Scientific) 24 to 48 hours before the IR and indicated assays. Plasmid sequences were verified using the T7 promoter sequencing primer, and expression levels were confirmed by immunoblotting for L2HGDH and detection of Myc or GFP tags. For KDM4B siRNA knockdown, H460 WT and LIPT1^−/−^ cells were transfected with 25 nM SMARTpool siGENOME Human KDM4A siRNA (M-004292-01, Horizon), SMARTpool siGENOME Human KDM4B siRNA (M-004290-01, Horizon), or siGENOME nontargeting siRNA pool as a control. siRNA was transfected using Lipofectamine 3000 transfection reagent (Thermo Fisher Scientific) 24 to 48 hours before the IR and indicated assays. Knockdown efficiency was confirmed by immunoblotting.

### Clonogenic survival analysis

Cells were plated in 60-mm dishes at a density ranging from 200 to 2000 cells per dish. Subsequently, the cells were subjected to increasing doses of IR (0 to 8 Gy) in triplicates for each treatment. After an incubation period of 10 to 14 days, the colonies were fixed using 6% glutaraldehyde and stained with 0.5% crystal violet solution. Colonies containing more than 50 cells were counted and quantified. The surviving fraction was then normalized to the corresponding sham control. Survival fraction curves were fitted using the linear-quadratic model by GraphPad Prism version 9.5.1 (La Jolla, CA). For α-KG rescue experiments, cells were pretreated with 1 mM dimethyl- α-KG or α-KG (MilliporeSigma) 24 hours before radiation.

### Xenograft experiments

Ten- to 12-week-old female athymic homozygous nude mice (Taconic, Germantown, NY) and C57BL/6J mice (The Jackson Laboratory, Bar Harbor, ME) were used for xenograft experiments. Xenografts were established by subcutaneously inoculating with 1 × 10^6^ WT and LIPT1^−/−^ H460 cells or KP9-3 cells into the flank. Once the tumors reached approximately 100 to 150 mm^3^ in volume, the mice were randomly assigned to specific treatment groups. A single dose of 10 Gy was delivered to the tumor site using the X-RAD 320 irradiator (Precision X-Ray Inc., North Branford, CT) (*77*). For CPI-613 treatment, nude mice received oral administration of vehicle control (5% DMSO, 30% PEG300, 10% Tween 80, and 55% water) or CPI-613 920 mg/kg) reconstituted in the same vehicle daily for 14 days, while C57BL/6 mice received oral treatment of vehicle control or CPI-613 (20 mg/kg) every other day for eight doses (the decreased dosing regimen was used because of increased lethargy and decreased activity after CPI-613 in C57BL/6 mice). Tumor sizes were measured three times per week, and tumor volume was calculated using the formula (L × W^2^)/2. Tumors were harvested for immunohistochemical (IHC) staining, and volume curves were continuously monitored until reaching the end point. Data are expressed as means ± SEM. Tumor volume and survival fraction curves were fitted using the logistic growth model by GraphPad Prism version 9.5.1. Blinding was used during data collection and analysis to minimize bias. Tumor measurements were conducted by researchers who were unaware of the treatment assignments. Animals with tumor volumes that did not reach the inclusion threshold of 100 to 150 mm^3^ at the start of treatment or that exhibited unrelated health concerns were excluded from the study. Similarly, animals that reached humane end points unrelated to tumor burden during the experimental period were excluded from analysis. All experimental procedures were conducted in compliance with the guidelines outlined by the Institutional Animal Care and Use Committee (protocol 2016-101694) and UT Southwestern institutional guidelines for animal care and ethics, adhering to the principles of the welfare and use of animals in cancer research. All values from the xenograft experiments are listed in data S2.

### IHC staining

Tumors were harvested and fixed in 10% formalin, followed by embedding in paraffin. Sections of 5-μm thickness were then deparaffinized and rehydrated, with antigen retrieval achieved using a 10 mM citrate buffer solution (pH 6.0) in a 95°C water bath for 30 min. Endogenous peroxidase activity was quenched using 10% hydrogen peroxide for 10 min. Subsequently, sections were blocked with 10% normal goat serum for 1 hour at room temperature to minimize nonspecific binding. Primary antibodies against Ki67 (Ab15580, RRID:AB_443209, dilution 1:2000, Abcam, Cambridge, UK) and γH2AX (2577, RRID:AB_2118010, dilution 1:200, Cell Signaling Technology) were applied overnight at 4°C. Following washing, sections were incubated with goat anti-rabbit biotinylated secondary antibodies (BA-1000, RRID:AB_2313606, dilution 1:2000, Vector Laboratories, Newark, CA) for 1 hour at room temperature. Signal amplification was achieved using an avidin/biotin ABC-HRP solution (Vector Laboratories), and antibody binding was visualized using 3,3′-diaminobenzidine (DAB) substrate (Vector Laboratories), with counterstaining performed using hematoxylin (Vector Laboratories). Last, the slides were dehydrated and coverslipped with mounting medium (Vector Laboratories). Imaging was conducted using a Keyence BZ-X700 All-in-one Fluorescence microscope (Keyence, Osaka, Japan), and DAB-positive nuclei signals were quantified in the tumor area and correlated with hematoxylin-stained nuclei using QuPath (*78*).

### Immunofluorescence staining

For immunofluorescence staining of γH2AX, RAD51, and H3K9me3, cells were seeded onto coverslips in six-well plates and allowed to adhere overnight. Following exposure to 0- to 4-Gy IR, the cells were fixed with 4% paraformaldehyde (PFA) for 10 min at room temperature and permeabilized with 0.5% Triton X-100 for 10 min. To visualize phosphorylated ATM (pATM-S1981), the cells were pre-extracted with cytoskeleton buffer [10 mM HEPES (pH 7.4), 300 mM sucrose, 100 mM NaCl, 3 mM MgCl2, and 0.1% Triton X-100] for 5 min post-IR followed by fixation and permeabilization. Subsequently, the cells were blocked with 4% BSA in phosphate-buffered saline (PBS) for 1 hour at room temperature to reduce nonspecific binding. Primary antibodies against γH2AX (05-636, RRID:AB_309864, dilution 1:500, MilliporeSigma), tri-methyl-Histone H3 (Lys9) (13969, RRID:AB_2798355, dilution 1:400, Cell Signaling Technology), tri-methyl-histone H3 (Lys4) (9751, RRID:AB_2616028, dilution 1:200, Cell Signaling Technology), tri-methyl-histone H3 (Lys27) (9733, RRID:AB_2616029, dilution 1:800, Cell Signaling Technology), Myc tag (2278, RRID:AB_490778, dilution 1:200, and 2276, RRID:AB_331783, dilution 1:1000, Cell Signaling Technology), pATM-S1981 (ab81292, RRID:AB_1640207, dilution 1:250, Abcam), and RAD51 (8875, RRID:AB_2721109, dilution 1:500, Cell Signaling Technology) were added to the cells and incubated overnight at 4°C. After washing with PBS, the cells were incubated with a fluorescently labeled secondary antibody for 1 hour at room temperature. Nuclei were counterstained with Hoechst 33342 or 4′,6-diamidino-2-phenylindole for 5 min. Last, coverslips were mounted onto glass slides using antifade mounting medium (Vector Laboratories).and sealed with nail polish. Images were captured using a Keyence BZ-X700 All-in-one Fluorescence microscope (Keyence), and the number of γH2AX and RAD51 foci per nucleus and the colocalization of ATM-pS1981 and γH2AX were quantified. Each experimental condition was assessed using two to three biological replicates with 60 to 100 cells analyzed per replicate.

### Neutral comet assay

The neutral comet assay was conducted following the manufacturer’s instructions (R&D Systems, Minneapolis, MN). Initially, cells were harvested at specified time points post 10-Gy IR and then embedded in low–melting point agarose, which was subsequently spread onto microscope slides to form a thin layer. After solidification, the slides were immersed in ice-cold lysis buffer for 1 hour at 4°C, followed by a wash and immersion in 1× tris-borate-EDTA (TBE) buffer for 15 min. Electrophoresis was then performed in a horizontal gel electrophoresis apparatus filled with freshly prepared 1× TBE for 30 min at 23 volts. Next, the slides underwent a series of washes with dH_2_O and 70% ethanol, followed by drying at 37°C. DNA fragments were stained using SYBR Gold Nucleic Acid Gel Stain (Thermo Fisher Scientific) in tris-EDTA buffer and visualized using a Keyence BZ-X700 All-in-one Fluorescence microscope (Keyence). Quantification of DNA damage was achieved by measuring parameters such as tail moment, which is calculated as the product of the tail length and the percentage of DNA in the comet tail (Tail moment = tail length × % of DNA in the tail). This analysis was performed using OpenComet (v1.3), a plugin for the image processing program ImageJ (*79*). Each experimental condition was assessed using two to three biological replicates with more than 100 cells analyzed per replicate.

### Metaphase chromosome spread assays

For the chromosome spread assays, cells were treated with colcemid (100 ng/ml; Irvine Scientific, Santa Ana, CA, USA) for 6 hours and harvested at 24 hours post 4-Gy IR, as previously described (*80*). Subsequently, the cells were hypotonically swollen in prewarmed 0.075 M KCl for 13 min at 37°C. Following hypotonic treatment, the cells were fixed by directly adding 1 ml of freshly made Carnoy’s fixative solution (methanol: acetic acid 3:1) into the hypotonic cell suspension for 5 min. The fixed cells were then dropped onto warmed glass slides and allowed to dry overnight. The slides with spread cells were stained with 5% Giemsa for 15 min at room temperature, gently rinsed with running water, air-dried, and mounted. Last, the slides were visualized using a microscope (BX51, Olympus, Tokyo, Japan). Chromosome aberrations, such as breaks, deletions, and chromatid interchanges were quantified (*28*). Each experimental condition was assessed using two to three biological replicates with more than 60 cells analyzed per replicate.

### Targeted metabolomics

For metabolomics analysis, three to four biological replicates of each cell line and treatment were used. Subconfluent cells cultured in a 10-cm plate were rinsed with saline and subsequently lysed with ice-cold 80% liquid chromatography–mass spectrometry (LC-MS)–grade methanol containing 0.1% formic acid in water. Following three freeze-thaw cycles in liquid nitrogen, the lysate underwent centrifugation to remove debris, and the resulting supernatant was collected and dried in a SpeedVac. The metabolite pellets were subjected to targeted metabolomics using quadrupole time-of-flight (Q-TOF) MS, as previously described (*81*). Briefly, this analysis was conducted using a 1290 UHPLC LC system interfaced with a high-resolution mass spectrometer (HRMS) 6550 iFunnel Q-TOF mass (Agilent Technologies, Santa Clara, CA), operating in both positive and negative ionization modes. For chromatographic separation, analytes were resolved on an Acquity UPLC HSS T3 column using a gradient elution with mobile phase A (0.1% formic acid in water) and mobile phase B (0.1% formic acid in 100% acetonitrile). The mass spectrometer source conditions were optimized for efficient ionization, and data acquisition was performed over the full mass/charge ratio range of 40 to 1700 in both positive and negative modes. Raw data files were processed using Profinder B.08.00 SP3 software (Agilent Technologies), leveraging an in-house database containing retention time and accurate mass information on 600 standards from the Mass Spectrometry Metabolite Library (IROA Technologies, SEA GIRT, NJ). Peak integration results obtained from Profinder were manually curated to ensure enhanced consistency. Subsequently, the peak area for each detected metabolite was normalized against the total ion count (TIC) of each sample, and these normalized areas were used as variables for multivariate and statistical analyses. Pathway enrichment analysis of differential metabolites among groups was conducted using Metaboanalyst 6.0 (*82*). The relative abundance of TCA cycle metabolites and 2HG in LIPT1^−/−^ H460 cells were calculated relative to the TIC normalized values of WT H460 cells. Details of TIC normalized metabolomics data are listed in data S3 and S4.

### Measurement of L-2HG

Metabolites were extracted from the samples with 80% methanol-water solution, and the resulting supernatant was dried in a SpeedVac. The dried pellet was mixed with [U-^13^C]L-2HG (internal standard for unlabeled samples, Cambridge Isotope Laboratories, 10 ng in 10 μl of acetonitrile). The mixture was dissolved in a diacetyl-l-tartaric anhydride (DATAN) solution (90 μl, 50 mg/mL in freshly mixed 80% acetonitrile/20% acetic acid, DATAN, Acros Organics). The solution was sonicated, warmed up to 75°C and kept for 30 min, cooled to room temperature, and then centrifuged again to collect the supernatant. The supernatant was again dried with a SpeedVac, and the pellet was reconstituted into 1.5 mM ammonium format aqueous solution with 10% acetonitrile (100 μl). LC-MS analysis was performed on an AB Sciex 5500 QTRAP LC-MS (Applied Biosystems SCIEX) equipped with a triple quadrupole/ion trap mass spectrometer with electrospray ionization interface and controlled by AB Sciex Analyst 1.6.1 software. Waters Acquity UPLC HSS T3 column (150 mM by 2.1 mM, 1.8 μM) column was used for separation. Solvents for the mobile phase were 1.5 mM ammonium formate aqueous (pH 3.6) adjusted with formic acid (A) and pure acetonitrile (B). The gradient elution was as follows: 0 to 12 min, linear gradient 1 to 8% B, and 12 to 15 min, 99% B, and then, the column was washed with 99% B for 5 min before reconditioning it for 3 min using 1% B. The flow rate was 0.25 ml/min, and the column was operated at 35°C. Multiple reaction monitoring was used to check 2-hydroxyglutarate-diacetyl tartrate derivatives: 363/147 (M + 0, CE: −14 V); 368/152 (M + 5, CE: −14 V). The relative abundance of L-2HG was normalized to the protein content of the cell pellet, which was dissolved in 0.1 N NaOH, and calculated relative to the treatment control.

### In situ PLA

Cells were initially harvested and fixed using 4% PFA, followed by permeabilization with 0.5% Triton X-100. An in situ PLA was conducted using Duolink PLA reagents (MilliporeSigma) in accordance with the manufacturer’s instructions. Before probing with primary antibodies, the cells were blocked with 4% BSA in PBS for 1 hour. Primary antibodies against histone H3 (819411, RRID:AB_2820127, dilution 1:200, BioLegend, San Diego, CA), TIP60 (NBP2-24613, RRID:AB_3272659, dilution 1:200, Novus Biologicals, Centennial, CO), γH2AX (05-636, RRID:AB_309864, dilution 1:200, MilliporeSigma), and ATM (NB100-309, RRID:AB_2243346, dilution 1:200, Novus Biologicals) in Duolink antibody diluent buffer were then added to the cells and incubated for 3 hours at room temperature. Subsequently, the cells were incubated with oligonucleotide-conjugated secondary antibodies (PLA probe anti-rabbit PLUS and anti-mouse MINUS), followed by ligation and amplification with a fluorophore-labeled oligonucleotide probe (DUO92014). Cell nuclei were counterstained with Hoechst 33342, and fluorescent images were captured using a Keyence BZ-X700 All-in-one Fluorescence microscope. Sites of PLA interaction were visualized as green dots, and the number of interactions was calculated and quantified for each treatment. Each experimental condition was evaluated using two to three biological replicates, with more than 100 cells analyzed per replicate.

### Subcellular fractionation

To examine the association of DNA repair proteins with chromatin post-IR, we used the subcellular protein fractionation kit (Thermo Fisher Scientific) following the manufacturer’s instructions. Briefly, the cells harvested at 0.5 hours post 10-Gy IR were lysed using cytoplasmic extraction buffer and membrane extraction buffer. Subsequently, the pellets were incubated with nuclear extraction buffer (NEB) to obtain the nuclear soluble lysate. The pellets were then subjected to incubation with NEB containing micrococcal nuclease and CaCl_2_ to isolate chromatin-bound proteins. The protein lysates obtained from these fractions were analyzed using gel electrophoresis and immunoblot analysis.

### Immunoblot analysis

Whole cell and tumor lysates were harvested and extracted with radioimmunoprecipitation assay buffer (MilliporeSigma) supplemented with protease and phosphatase inhibitor (MilliporeSigma) and then incubated on ice for 20 min. After centrifugation to clear the lysate, the protein concentration of each sample was measured using the Pierce bicinchoninic acid protein assay kit (Thermo Fisher Scientific). SDS–polyacrylamide gel electrophoresis was performed using 20 μg of protein, and the separated proteins were transferred onto the nitrocellulose membrane (Bio-Rad). The membranes were blocked using non-fat milk in tris-buffered saline containing 0.1% Tween-20 (Bioworld, Dublin, OH) and then incubated with primary antibodies overnight at 4°C. The nitrocellulose-bound primary antibodies were washed and incubated with horseradish peroxidase–linked secondary antibodies (Cell Signaling Technology). The immunoblots were reacted using Pierce enhanced chemiluminescent Western Blotting Substrate (Thermo Fisher Scientific), exposed to x-ray films, and then developed using a Protec x-ray film processor.

We used the following primary antibodies: anti-LA (437695, RRID:AB_212120, Millipore), anti-DLAT (12362, RRID:AB_2797893, Cell Signaling Technology), anti-DLST (5556, RRID:AB_10695157, Cell Signaling Technology), anti-tubulin (T5168, RRID:AB_477579, Sigma-Aldrich), anti–glyceraldehyde-3-phosphate dehydrogenase (8884, RRID:AB_11129865, Cell Signaling Technology), anti-ATM (2873, RRID:AB_2062659, Cell Signaling Technology), anti–p-ATM (S1981) (Ab81292, Abcam), anti-Chk2 (6334, RRID:AB_11178526, Cell Signaling Technology), anti–p-Chk2 (Thr^68^) (2197, RRID:AB_2080501, Cell Signaling Technology), anti-γH2AX (05-636, MilliporeSigma), anti-Ku70 (sc-17789, Santa Cruz Biotechnology), anti–histone H3 (4499, RRID:AB_10544537, Cell Signaling Technology), anti-L2HGDH (15707-1-AP, RRID:AB_2133202 Proteintech), anti-KDM4A (5328, RRID:AB_10828595, Cell Signaling Technology), anti-KDM4B (8639, RRID:AB_11140642, Cell Signaling Technology), anti–Myc tag (2276, RRID:AB_331783, Cell Signaling Technology), and anti–acetyl-lysine Antibody (06-933, RRID:AB_310304, MilliporeSigma).

### Laser micro-IR and live cell imaging

The GFP-EXO1 plasmid, provided by A.J.D., was transfected into WT and LIPT1^−/−^ H157 cells using Lipofectamine 3000 transfection reagent (Thermo Fisher Scientific) in 35-mm glass-bottom culture dishes (MatTek Cultureware, Ashland, MA). Twenty-four hours posttransfection, laser micro-IR and real-time recruitment experiments were conducted using a Carl Zeiss Axiovert 200 M microscope equipped with a Plan-Apochromat 63×/numerical aperture 1.40 oil immersion objective, as previously described (*83*, *84*). A 365-nm pulsed nitrogen laser (Spectra-Physics, Milpitas, CA) was directly coupled to the epifluorescence path of the microscope and operated at 80% laser output at 10 Hz for 400 ms to generate a consistent number of micro (DSBs) in each experiment. Time-lapse images were captured using a Carl Zeiss AxioCam HRm camera while cells were maintained in CO_2_-independent medium (Invitrogen) at 37°C. Fluorescence intensities of the micro-irradiated area and control area were quantified using ImageJ software, and the intensity of irradiated areas was normalized to nonirradiated control areas, as previously described (*85*). To account for nonspecific photobleaching, background fluorescence was subtracted from the accumulation spot. Relative fluorescence intensity (RF) was calculated using the formula: RF(t) = (It − IpreIR)/(Imax − IpreIR), where IpreIR represents the fluorescence intensity of the micro-irradiated area before IR, and Imax represents the maximum fluorescence signal in the micro-irradiated area. Fold increase (FI) was calculated using the formula: FI(t) = It/IpreIR. Each experimental condition was assessed using three biological replicates, with 10 independent measurements.

### HR reporter assay

The DR-GFP HR HEK293 cell lines with single insertions, confirmed by Southern hybridization, were provided by J. M. Stark and B. Chen (*86*). LIPT1 CRISPR knockout was performed in the DR-GFP HR stable cell lines. HR assays were conducted as previously described (*49*) by transiently transfecting 2.5 μg of the I-SceI enzyme plasmid to initiate the assay. In addition, 0.1 μg of DsRed plasmid was cotransfected with I-SceI to control for potential alterations in transfection efficiency or survival. Four days after transfection, cells undergoing HR successfully expressed GFP, which was detected by Amnis FlowSight imaging flow cytometry (Luminex, Austin, TX). The percentage of HR activity was assessed by quantifying the population of cells positive for both DsRed and GFP. Each experimental condition was evaluated using three biological replicates.

### Statistics and reproducibility

Statistical details including *n*, mean, and statistical significance values are indicated in the text, figure legends, or methods. The error bars in the experiments represent the SEM or SD from either independent experiments or independent samples. All statistical analyses were performed in Prism v.9.5.1 (GraphPad Software); detailed information about the statistical methods used, such as *t* test, one-way, or two-way analysis of variance (ANOVA), is specified in the figure legends or methods. The numbers of independent experiments or biological replicate samples and *P* values (*P < 0.05, **P < 0.01, ***P < 0.001, ****P < 0.0001) are provided in the individual figures. *P* < 0.05 was considered statistically significant. Figures 1, 2, 4, 5, 6, 7, 8, 9, and 10 and figs. S1, S2, S3, S4, S5, S6, S7, and S8 show representative images of at least three independent experiments or biological replicate samples with similar results.
