# Supplementary material for: Lipoylation inhibition enhances radiation control of lung cancer by suppressing homologous recombination DNA damage repair
Source: Sci Adv. 2025 Mar 12;11(11):eadt1241. doi: 10.1126/sciadv.adt1241 (PMC11900879; doi:10.1126/sciadv.adt1241)
Supplement: Supplementary file 1 — Figs. S1 to S8 Legends for data S1 to S4 [file sciadv.adt1241_sm.pdf]

Science Advances
AAAS

Supplementary Materials for

Lipoylation inhibition enhances radiation control of lung cancer by suppressing homologous recombination DNA damage repair

Jui-Chung Chiang *et al.*

Corresponding author: Ralph J. DeBerardinis, ralph.deberardinis@utsouthwestern.edu;
Yuanyuan Zhang, yuanyuan.zhang@utsouthwestern.edu

Sci. Adv. **11**, eadt1241 (2025)
DOI: 10.1126/sciadv.adt1241

The PDF file includes:

Figs. S1 to S8
Legends for data S1 to S4

Other Supplementary Material for this manuscript includes the following:

Data S1 to S4

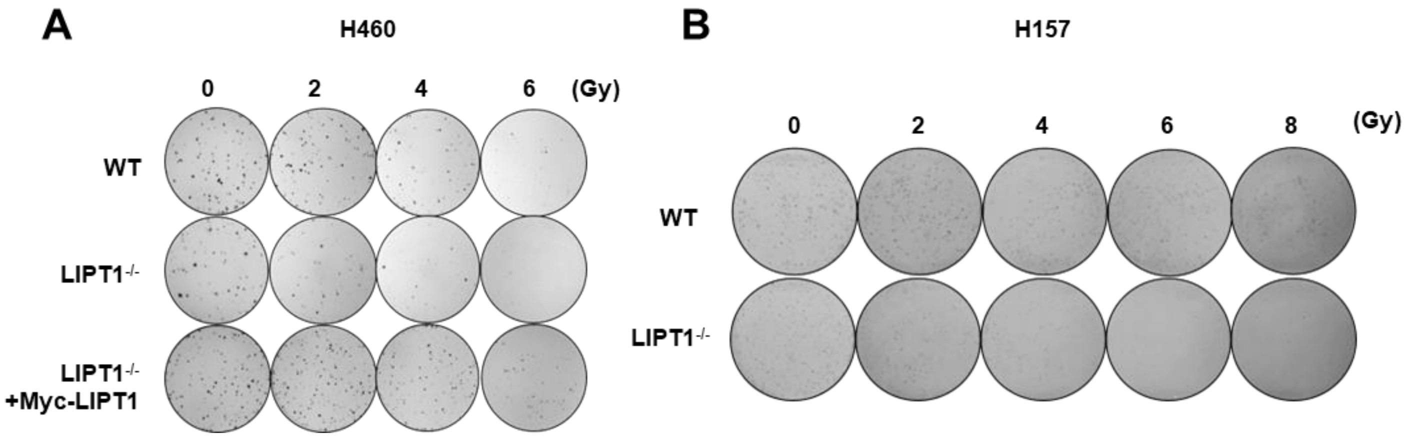

Fig. S1. Clonogenic assays of human non-small cell lung cancer cell lines at varying doses of irradiation. (**A-B**) Representative images of colony formation in indicated cell lines in H460 (A) and H157 (B) subjected to various doses of irradiation.

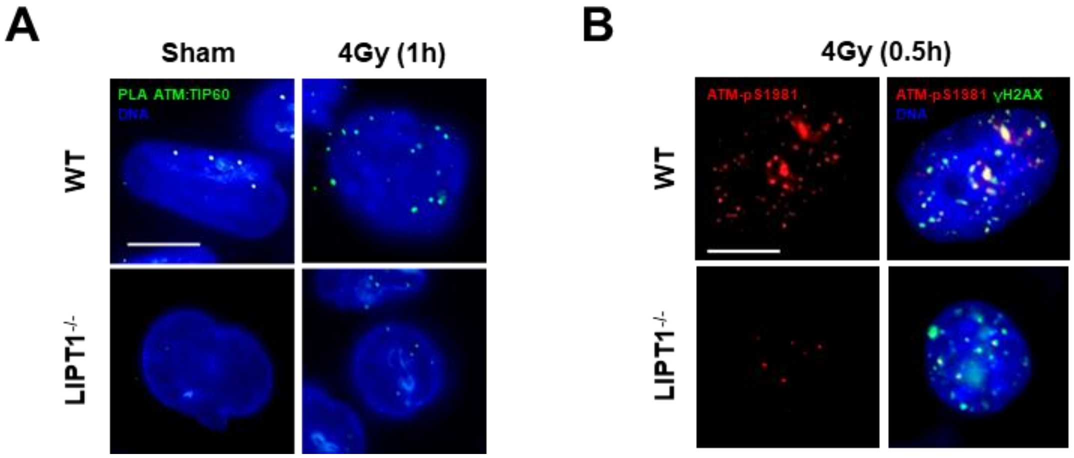

Fig. S2. LIPT1$^{-/-}$ H157 exhibits decreased interaction of ATM and TIP60 interaction and ATM phosphorylation. (**A**) Representative images of in situ proximity ligation assay (PLA, green dots) of ATM and TIP60 interaction in WT and LIPT1$^{-/-}$ H157 cells with or without radiation. Nuclei were stained with Hoechst 33342. Scale bar, 10 μm. (**B**) Representative images of ATM-pS1981 (Red) and γH2AX (Green) by immunofluorescence staining cells at 0.5 hours after 4Gy. Nuclei were stained with DAPI. Scale bar, 10 μm.

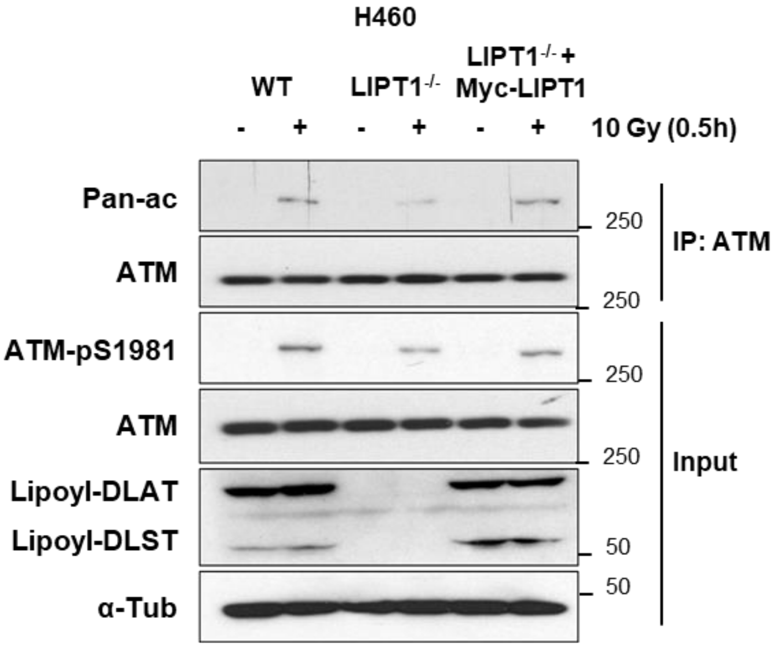

Fig. S3. LIPT1$^{-/-}$ H460 cells exhibit decreased ATM acetylation. ATM acetylation was assessed via immunoprecipitation (IP) using a Pan-Ac-Lysine antibody (Pan-ac) targeting ATM pull-down complexes. Acetylation signals were detected at the molecular weight corresponding to ATM. α-Tubulin served as the loading control.

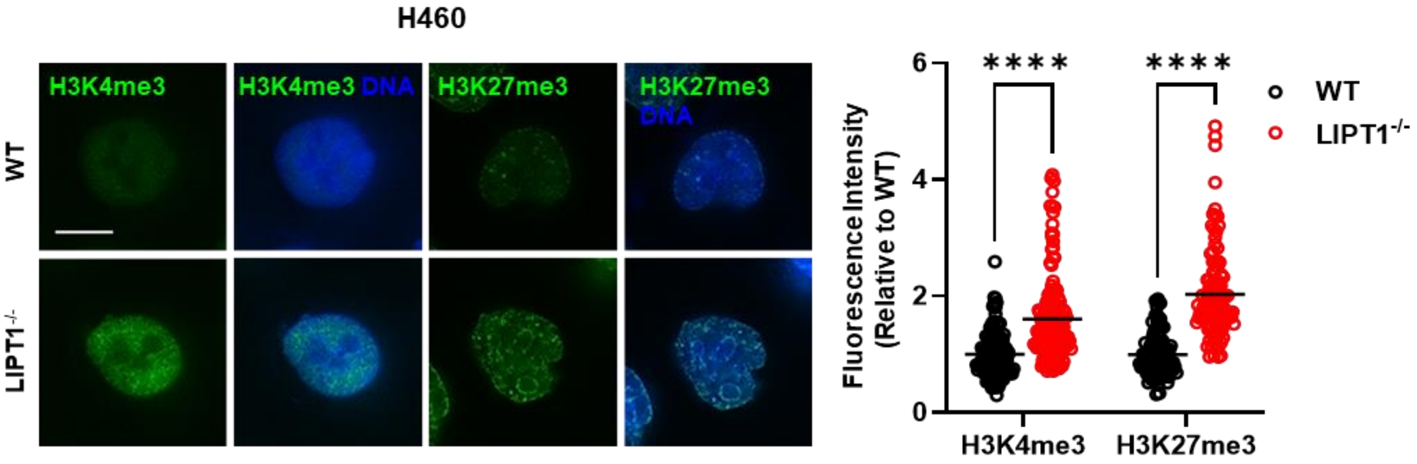

Fig. S4. LIPT1$^{-/-}$ cells exhibit elevated baseline H3K4me3 and H3K27me3. Representative images and quantification of H3K4me3 and H3K27me3 by immunofluorescence staining in WT and LIPT1$^{-/-}$ H460 cells. Nuclei were stained with Hoechst 33342. Scale bar, 10 µm. Imaging and quantification were performed on >100 cells per treatment. Two-way ANOVA was used for the statistical analyses. ****p < 0.0001.

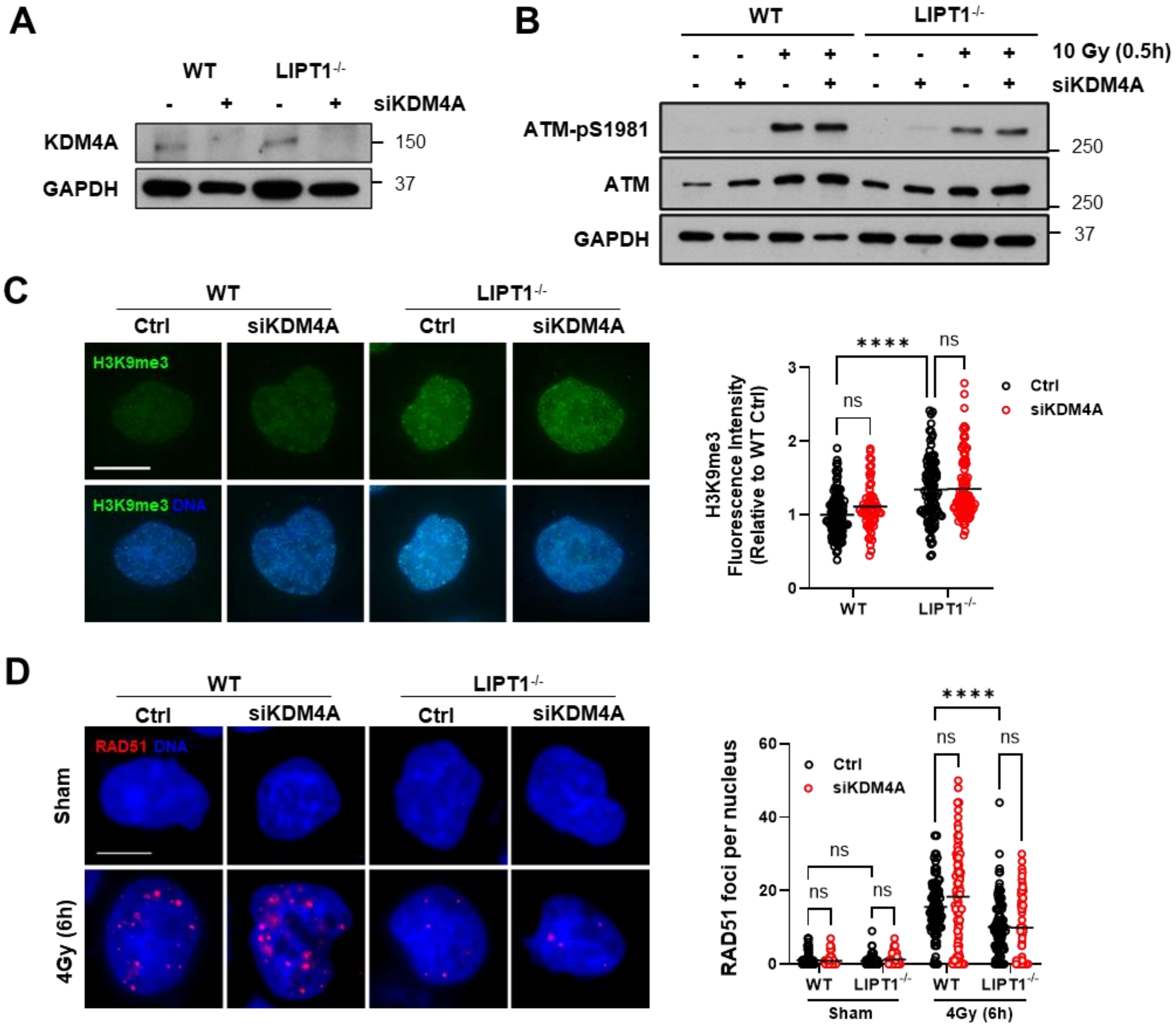

Fig. S5. KDM4A knockdown has no effect on H3K9me3 level and RAD51 recruitment. (**A**) Representative immunoblots validating siRNA suppression of KDM4A in WT and LIPT1$^{-/-}$ H460 cells, with GAPDH used as a loading control. **(B)** Immunoblotting analysis of pS1981 and total ATM in Ctrl and siKDM4A WT and LIPT1$^{-/-}$ H460 cells, with GAPDH used as a loading control. **(C)** Representative images and quantification of H3K9me3 by immunofluorescence staining in Ctrl and siKDM4A WT and LIPT1$^{-/-}$ H460 cells. **(D)** Representative images and quantification of RAD51 foci by immunofluorescence staining in nonirradiated control and at 6 hours after 4Gy in Ctrl and siKDM4A WT and LIPT1$^{-/-}$ H460 cells. Nuclei were stained with Hoechst 33342. Scale bar, 10 μm. Imaging and quantification were performed on >100 cells per treatment. Two-way ANOVA was used for the statistical analyses. ****p < 0.0001.

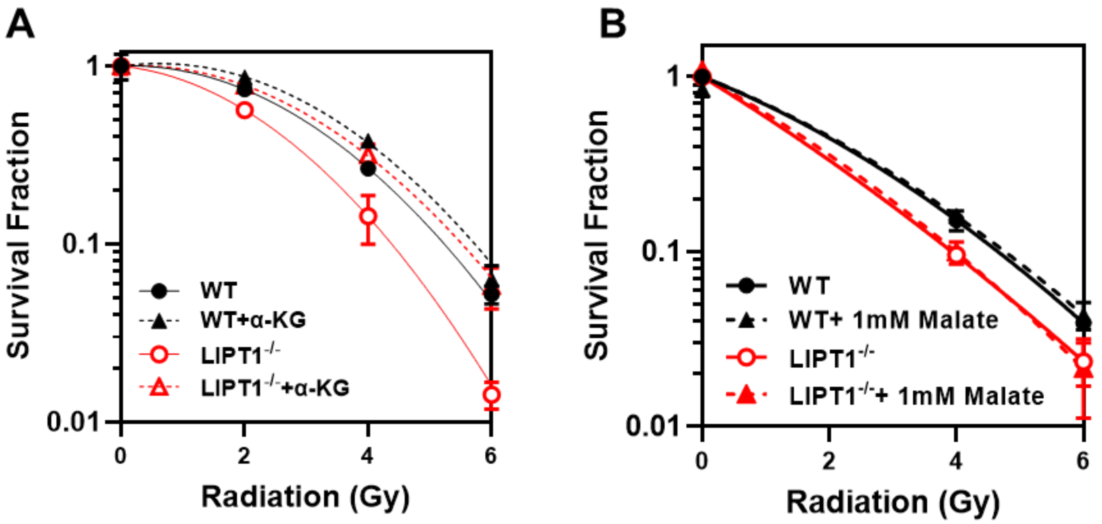

Fig. S6. α-KG, but not malate, enhances survival of LIPT1$^{-/-}$ H460 after radiation. (**A, B**) Clonogenic assay of WT and LIPT1$^{-/-}$ H460 cells with or without 1 mM αKG (A) and 1 mM Malate (B) after 2, 4, 6 Gy. The surviving fraction was normalized to the corresponding sham control and survival curves were fitted using the linear-quadratic model.

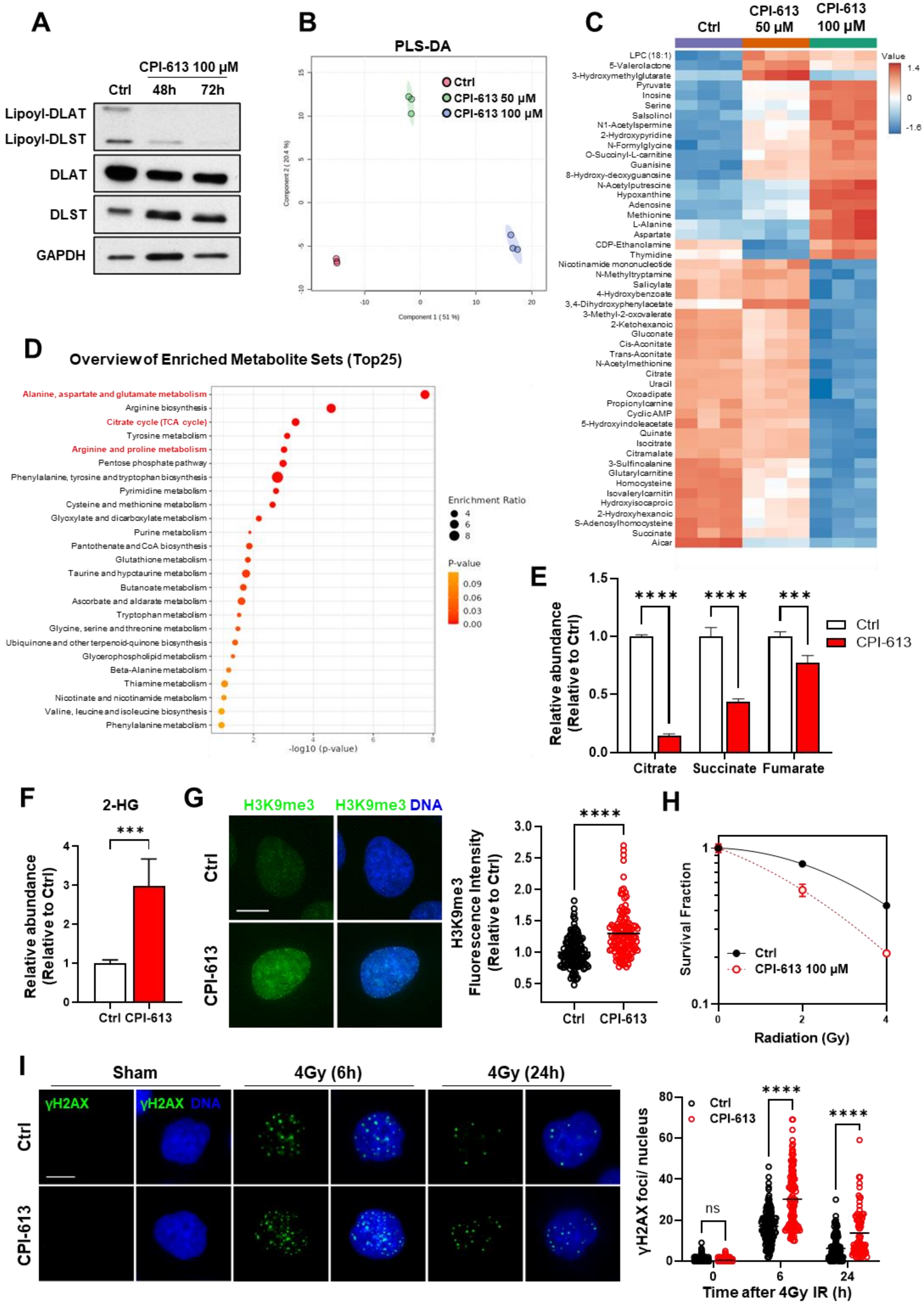
A
CPI-613 100 μM
Ctrl
48h
72h
Lipoyl-DLAT
Lipoyl-DLST
DLAT
DLST
GAPDH
B
PLS-DA
Ctrl
CPI-613 50 μM
CPI-613 100 μM
Component 1 (51 %)
Component 2 (20.4 %)
C
Ctrl
CPI-613 50 μM
CPI-613 100 μM
Value
1.4
0
-1.6
LPC (18:1)
5-Valerolactone
3-Hydroxymethylglutarate
Pyruvate
Inosine
Serine
Salsolinol
N1-Acetylspermine
2-Hydroxypyridine
N-Formylglycine
O-Succinyl-L-carnitine
Guanisine
8-Hydroxy-deoxyguanosine
N-Acetylputrescine
Hypoxanthine
Adenosine
Methionine
L-Alanine
Aspartate
CDP-Ethanolamine
Thymidine
Nicotinamide mononucleotide
N-Methyltryptamine
Salicylate
4-Hydroxybenzoate
3,4-Dihydroxyphenylacetate
3-Methyl-2-oxovalerate
2-Ketohexanoic
Gluconate
Cis-Aconitate
Trans-Aconitate
N-Acetylmethionine
Citrate
Uracil
Oxoadipate
Propionylcarnine
Cyclic AMP
5-Hydroxyindoleacetate
Quinate
Isocitrate
Citramalate
3-Sulfinoalanine
Glutarylcarnitine
Homocysteine
Isovalerylcarnitin
Hydroxyisocaproic
2-Hydroxyhexanoic
S-Adenosylhomocysteine
Succinate
Aicar
D
Overview of Enriched Metabolite Sets (Top25)
Alanine, aspartate and glutamate metabolism
Arginine biosynthesis
Citrate cycle (TCA cycle)
Tyrosine metabolism
Arginine and proline metabolism
Pentose phosphate pathway
Phenylalanine, tyrosine and tryptophan biosynthesis
Pyrimidine metabolism
Cysteine and methionine metabolism
Glyoxylate and dicarboxylate metabolism
Purine metabolism
Pantothenate and CoA biosynthesis
Glutathione metabolism
Taurine and hypotaurine metabolism
Butanoate metabolism
Ascorbate and aldarate metabolism
Tryptophan metabolism
Glycine, serine and threonine metabolism
Ubiquinone and other terpenoid-quinone biosynthesis
Glycerophospholipid metabolism
Beta-Alanine metabolism
Thiamine metabolism
Nicotinate and nicotinamide metabolism
Valine, leucine and isoleucine biosynthesis
Phenylalanine metabolism
-log10 (p-value)
Enrichment Ratio
4
6
8
P-value
0.09
0.06
0.03
0.00
E
Relative abundance (Relative to Ctrl)
Ctrl
CPI-613
Citrate
Succinate
Fumarate
****
****
***
F
2-HG
***
Relative abundance (Relative to Ctrl)
Ctrl
CPI-613
G
H3K9me3
H3K9me3 DNA
Ctrl
CPI-613
H3K9me3 Fluorescence Intensity (Relative to Ctrl)
****
H
Survival Fraction
Radiation (Gy)
Ctrl
CPI-613 100 μM
I
Sham
4Gy (6h)
4Gy (24h)
γH2AX
γH2AX DNA
Ctrl
CPI-613
γH2AX foci/ nucleus
ns
****
****
Time after 4Gy IR (h)

Fig. S7. CPI-613 recapitulates the metabolic and DNA damage phenotype of LIPT1$^{-/-}$ cells. (**A**) Immunoblotting analysis of total- and lipoyl-DLAT, total- and lipoyl-DLST in H460 cells treated with vehicle,100 µM CPI-613 for 48-72 hours. GAPDH is the loading control. (**B**) Partial Least Squares Discriminant Analysis (PLS-DA) of metabolomic profiles in H460 cells treated with vehicle, 50 µM and 100 µM CPI-613 for 48 hours. (**C**) Heatmap analysis of the top 50 differential metabolites in H460 cells treated with vehicle, 50 µM and 100 µM CPI-613 for 48 hours. (**D**) Metabolite set enrichment analysis comparing H460 cells treated with vehicle or 100uM CPI-613 for 48 hours. (**E**) Relative abundance of the indicated TCA cycle metabolites in vehicle or 100 µM CPI-613 cells. Data were represented as mean ± SD., two-way ANOVA was used for the statistical analyses. ***p < 0.001, ****p < 0.0001. (**F**) Relative abundance of 2-hydroxyglutarate (2HG) in vehicle or 100 µM CPI-613 cells. Unpaired t tests were used for the statistical analyses. ****p < 0.001. (**G**) Representative images and quantification of H3K9me3 by immunofluorescence staining in vehicle or 100 µM CPI-613 cells. Nuclei were stained with Hoechst 33342. Scale bar, 10 µm. Unpaired t tests were used for the statistical analyses. ****p < 0.0001. (**H**) Clonogenic assay of vehicle or 100 µM CPI-613 cells after 2 and 4 Gy. The surviving fraction was normalized to the corresponding sham control and survival curves were fitted using the linear-quadratic model. (**I**) Representative images and quantification of γH2AX foci by immunofluorescence staining in non-irradiated cells and at 6 h and 24 h after 4 Gy irradiation in vehicle and 100 µM CPI-613 cells. Nuclei were stained with Hoechst 33342. Scale bar, 10 µm. Two-way ANOVA was used for the statistical analyses. ****p < 0.0001.

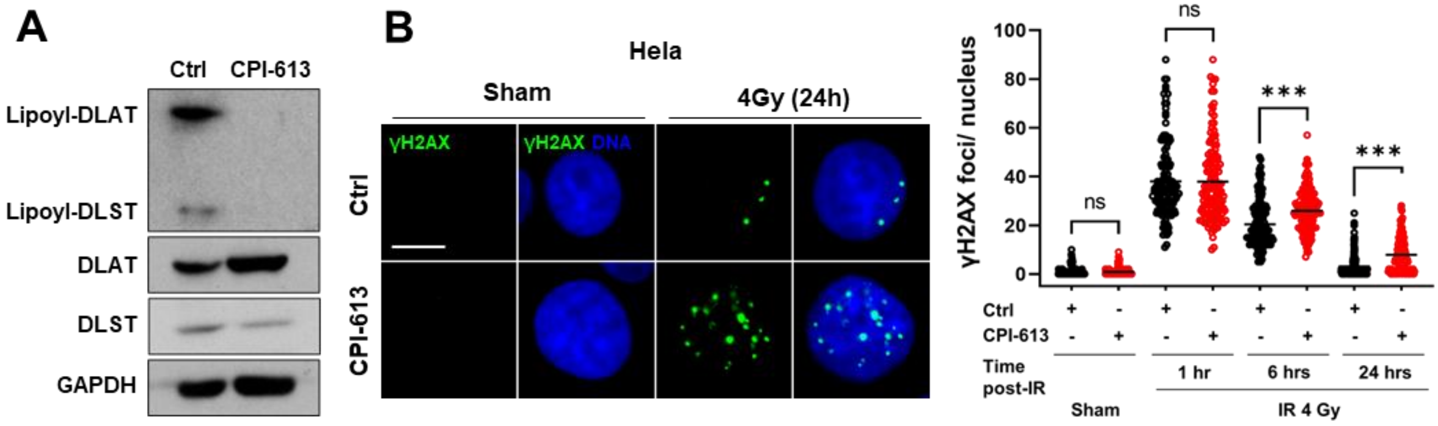

Fig. S8. CPI-613 inhibits the lipoylation of 2-ketoacid dehydrogenases and impairs IR induced DNA damage repair in HeLa cells. (**A**) Immunoblotting analysis of total- and lipoyl-DLAT, and total- and lipoyl-DLST in HeLa cells treated with 100 µM CPI-613 for 48 hours. GAPDH is the internal control. (**B**) Representative images and quantifications of γH2AX foci at specific time points after 4Gy by immunofluorescence staining in HeLa cells pretreated with DMSO or 100 µM CPI-613 for 48 hours. Nuclei were stained with Hoechst 33342. Scale bar, 10 µm. Imaging and quantification were performed on >100 cells per treatment. Two-way ANOVA was used for the statistical analyses. ***$p < 0.001$.

Supplementary data S1: Details for CRISPR library.
Supplementary data S2: All data values from xenograft experiments
Supplementary data S3: Total ion count normalized metabolomics data from WT and $LIPT1^{-/-}$ H460 cells
Supplementary data S4: Total ion count normalized metabolomics data from Ctrl and CPI-613 H460 cells
